# LB-CIM: Low-Bandwidth Cross-Modal Interaction for Multimodal Visible-SAR Object Detection

**DOI:** 10.3390/s26217078

**Published:** 2026-11-09

**Authors:** Yang Li, Xinrong Wang, Kejia Zhang, Xiangyu Peng

**Affiliations:** 1 College of Computer Science and Technology, Harbin Engineering University, Harbin 150001, China; kejiazhang@hrbeu.edu.cn; 2 Harbin Space Star Data System Technology Co., Ltd., Harbin 150028, China; 3 School of Earth and Space Science and Technology, Wuhan University, Wuhan 430072, China; 19520041508@163.com; 4 Space Star Technology Co., Ltd., Beijing 100095, China; 5 Faculty of Information Technology, Beijing University of Technology, Beijing 100124, China; s202487421@emails.bjut.edu.cn

**Keywords:** multimodal sensing, visible-SAR object detection, multi-sensor data fusion, cross-modal interaction, multimodal representation learning, remote sensing, low-bandwidth attention

## Abstract

Visible and synthetic aperture radar (SAR) sensors provide heterogeneous yet complementary observations for remote-sensing object detection. However, effective multimodal sensing fusion remains challenging because SAR features may contain locally unstable responses, while dense cross-modal interaction can introduce redundant information exchange and unnecessary computation. This paper presents LB-CIM, a low-bandwidth cross-modal interaction module for paired VIS–SAR object detection. LB-CIM adopts a three-stage design. First, multi-scale consistency denoising (MCD) estimates local SAR instability from cross-scale discrepancies and suppresses inconsistent responses before fusion. Second, differential low-bandwidth cross-attention (DLBCA) performs bidirectional interaction using queries at the unreduced interaction-grid resolution and spatially reduced key–value representations, while differential values and difference-aware gating emphasize non-redundant cross-modal information. Third, consensus-complementary enhancement (CCE) separates shared and complementary cues and adaptively reorganizes them to form the fused representation. LB-CIM is inserted at multiple feature levels of a dual-branch detector. Experiments on OGSOD-1.0 and SpaceNet6-OTD-Fog demonstrate consistent improvements over single-modal and multimodal baselines. On OGSOD-1.0, the proposed model achieves 95.1% mAP_50_ and 56.8% mAP_50:95_, improving the YOLO11-RGBT baseline by 4.8 and 4.7 percentage points, respectively. On SpaceNet6-OTD-Fog, it reaches 70.6% mAP_50_ and 38.5% mAP_50:95_, corresponding to gains of 2.7 and 2.0 percentage points. Ablation experiments confirm the complementary contributions of MCD, DLBCA, and CCE, while evaluations on YOLOv5n, YOLOv8n, and YOLO11n-based detectors show that the gains are maintained across different YOLO architectures. The complete model contains 4.6 M parameters and requires 10.4 GFLOPs, providing a favorable balance between detection accuracy and computational complexity. Qualitative comparisons further show fewer missed detections and false positives under diverse target densities and scene structures. These results demonstrate that LB-CIM provides an effective and computationally efficient solution for multimodal sensing representation learning and VIS–SAR information fusion in remote-sensing vision tasks.

## 1. Introduction

Object detection in remote sensing imagery provides spatially explicit information for applications such as maritime surveillance, urban monitoring, infrastructure inspection, and disaster assessment. Deep neural detectors have substantially improved the recognition and localization of targets in optical remote sensing images, where spectral appearance, texture, and fine spatial details are usually informative for distinguishing man-made objects from surrounding backgrounds [1,2]. Nevertheless, visible (VIS) imagery remains dependent on illumination and atmospheric conditions, and its discriminative cues can be weakened by cloud cover, haze, shadows, or low contrast. Synthetic aperture radar (SAR) offers a complementary sensing mechanism. As an active microwave imaging system, SAR supports day-and-night and weather-independent observation and is sensitive to geometric structure and scattering properties [3]. These advantages are accompanied by a different set of limitations. Coherent imaging introduces speckle, local responses can be spatially unstable, and the resulting intensity patterns are less directly related to optical texture and color [4,5]. Consequently, neither modality provides uniformly reliable evidence across diverse remote sensing scenes. From a multimodal sensing perspective, VIS and SAR provide heterogeneous yet complementary observations with distinct physical acquisition mechanisms. Joint VIS–SAR detection is therefore attractive because it can combine detailed appearance information from VIS imagery with structural and scattering cues from SAR, reducing the dependence of a detector on a single sensing condition.

Multimodal sensing data fusion in remote sensing has gradually moved from simple input combination toward feature-level interaction, where modality-specific representations from heterogeneous sensors can be retained before being selectively exchanged and aggregated [6]. Existing fusion strategies are commonly organized into data-level, feature-level, and decision-level approaches [7]. Data-level fusion combines source images before feature extraction and can make direct use of raw observations, but it is sensitive to registration errors and modality-specific interference. Decision-level fusion maintains independent processing streams until the prediction stage, which offers flexibility but leaves little opportunity for fine-grained cross-modal interaction. Feature-level fusion provides a more suitable compromise for object detection because semantic representations from heterogeneous sensing modalities can be jointly optimized with the detection objective [8]. A related distinction in multispectral detection is between “fusion-then-detection” pipelines and unified detectors. The former first generate a fused image and subsequently apply a single-modality detector, whereas the latter learn fusion and detection within the same network. Early feature-level approaches mainly relied on concatenation, addition, or relatively shallow feature interaction. YOLOrs introduced mid-level multimodal fusion into a detector designed for remote sensing imagery [9], while SuperYOLO combined compact multimodal fusion with auxiliary super-resolution learning to strengthen the representation of small targets [10]. These studies demonstrate the value of complementary sensing modalities, but direct aggregation does not explicitly distinguish useful modality-specific information from interference. When heterogeneous sensor representations are mixed without sufficient interaction or selection, redundant responses and noise can be propagated together with informative cues.

Recent studies have therefore placed greater emphasis on cross-modality learning through cross-modal attention, feature decomposition, and adaptive regulation. Transformer-based architectures provide a natural mechanism for modeling long-range dependencies across feature sequences [11]. Hierarchical and computationally efficient Transformer designs have further extended attention-based modeling to dense visual prediction while reducing the computational burden associated with global interaction [12,13,14]. Transformer-based feature modeling has also been increasingly adopted in remote sensing object detection to combine local spatial details with broader contextual information [15,16,17,18]. CFT introduced Transformer-based intra- and inter-modality interaction for multispectral detection [19], and ICAFusion further employed iterative dual cross-attention to capture complementary information while controlling the cost associated with repeatedly stacked interaction blocks [20]. Other methods explicitly model relationships between shared and modality-specific representations.Cross-modality attentive fusion has also been explored to selectively aggregate complementary representations from heterogeneous sensing modalities [21]. MMYFNet uses cosine-similarity-guided decomposition to separate common and specific features before fusion [22], whereas ACDF-YOLO introduces attentive cross-differential fusion to strengthen complementary feature modeling [23]. Complementarity-oriented Transformer designs have likewise been investigated in RGB–infrared detection to calibrate cross-modal interaction and emphasize mutually supportive information [24]. Asymmetric feature supplementation and scene-adaptive modality regulation have also been explored in optical-infrared and RGBT detection. Wang et al. designed a dual-branch asymmetric attention architecture in which one modality supplements information that is relatively weak in the other [25], while illumination-guided RGBT detection dynamically regulates inter- and intra-modality fusion according to scene conditions [26]. Related work on RGB-T salient object detection has also explored structured modality-specific and modality-complementary learning. Tang et al. proposed ConTriNet, which models these cues through a triple-flow architecture and integrates the resulting predictions using flow-cooperative fusion [27]. Benchmark construction and target-aware adversarial learning have also been investigated to improve visible–infrared detection across different multimodal scenarios [28]. Adaptive multimodal regulation has further been explored through prior-enhanced mixture-of-experts fusion, where modality contributions are dynamically coordinated according to feature characteristics [29]. EI^2^Det further establishes visible-priority and infrared-priority cross-attention and adjusts modality contributions according to illumination intensity [30]. These developments indicate that multimodal detection depends not only on combining two feature streams, but also on determining what information should interact and how the transferred information should be regulated. For the more heterogeneous VIS–SAR setting, YOLO-CMFM directly addresses cross-modal discrepancy through edge-guided processing [31], bidirectional cross-attention, and context-guided gating. More recently, correlation-guided progressive interaction and gated fusion have been investigated to progressively exchange and regulate information between optical and SAR representations [32]. The recent construction of optical-SAR benchmarks such as OGSOD-2.0 also reflects growing interest in learning joint representations from these two sensing modalities [33].

Although these methods demonstrate the effectiveness of adaptive multimodal interaction, their mechanisms emphasize different aspects of cross-modal fusion. ICAFusion employs iterative dual cross-attention to repeatedly aggregate complementary representations between modalities [20], whereas MMYFNet uses cosine-similarity-guided decomposition to separate common and modality-specific features before subsequent refinement and fusion [22]. ACDF-YOLO explicitly models cross-modal differences through a dedicated differential fusion module combined with attention-based feature enhancement [23]. In the VIS–SAR setting, YOLO-CMFM combines edge-guided processing, bidirectional cross-attention, and context-guided gating to regulate heterogeneous feature interaction [31], while correlation-guided progressive interaction uses cross-modal correlation priors together with gated fusion to adaptively control complementary information exchange [32]. These approaches mainly regulate cross-modal interaction through similarity decomposition, attention-guided retrieval, differential feature modeling, or modality-dependent weighting. In contrast, the proposed LB-CIM jointly considers the reliability of the SAR representation before interaction, the amount and content of information transferred during interaction, and the organization of agreement- and discrepancy-oriented responses after interaction. This distinction motivates the reliability–interaction–enhancement design described below.

Despite this progress, three issues remain particularly relevant to VIS–SAR multimodal sensing fusion. First, the reliability of the SAR representation should be considered before intensive cross-modal interaction. Local high-frequency responses in SAR features may arise from meaningful scattering structures, but they may also contain speckle-related or locally unstable components. Uniform smoothing risks weakening useful boundaries and structural responses, whereas directly forwarding all SAR activations into an interaction block allows unreliable components to influence the opposite modality. Therefore, the first problem is not simply whether SAR features should be filtered, but how potentially unstable responses can be selectively attenuated before they participate in cross-modal information exchange.

Second, existing cross-modal attention mechanisms mainly determine where information should be retrieved from the opposite modality, whereas the content being transferred is still commonly represented by the complete counterpart value features. For heterogeneous VIS and SAR representations, this may propagate information that is already similar across the two modalities together with modality-specific responses. Moreover, performing cross-modal matching over dense feature tokens at multiple pyramid levels increases the amount of interaction. A more selective interaction mechanism should therefore consider both the interaction bandwidth and the transferred content: the former determines how much information participates in cross-modal matching, while the latter determines what information is actually delivered to the receiving modality.

Third, cross-modal differences can be exploited in different ways. Existing similarity decomposition, differential fusion, and modality-weighting strategies mainly use cross-modal relationships to separate feature types, enhance salient differences, or assign relative modality importance. After bidirectional interaction, however, agreement-oriented and discrepancy-oriented responses may still coexist within the two updated modality features. Direct concatenation or a single modality-level gate does not explicitly regulate the relative contribution of these two response types. This motivates a post-interaction mechanism that characterizes cross-modal agreement and discrepancy separately and adaptively determines their contributions to the final fused representation.

Motivated by these observations, we develop a Low-Bandwidth Cross-modal Interaction Module (LB-CIM) for progressive VIS–SAR feature fusion. Rather than treating cross-modal fusion as a single aggregation operation, LB-CIM follows a reliability–interaction–enhancement sequence, with each stage addressing a different aspect of heterogeneous information transfer.

MCD operates on the SAR branch before cross-modal interaction. Instead of directly applying a low-pass filter, it combines a local residual cue with a cross-scale discrepancy cue to estimate an element-wise suppression response. The resulting response modulates a signed residual correction, allowing potentially unstable SAR activations to be selectively attenuated without uniformly smoothing the complete feature representation.

DLBCA subsequently performs bidirectional interaction between the refined SAR representation and the corresponding VIS feature. Both modalities are projected into a compact shared attention space, while the queries retain the full spatial resolution of the compact interaction grid and only the key/value representations are further spatially reduced. This design decreases the number of tokens participating in cross-modal matching. Unlike conventional cross-attention, which directly transfers the complete counterpart value representation, DLBCA constructs directional differences between spatially corresponding reduced value tokens and aggregates these differential values according to cross-modal attention. A local difference-aware gate further regulates the magnitude of the residual update. In this formulation, channel projection and spatial reduction control the interaction bandwidth, differential values determine the transferred content, and the gate regulates how strongly this information modifies the receiving modality.

Following bidirectional interaction, CCE further organizes the updated modality representations. Rather than assigning a single relative weight to the VIS and SAR branches, it constructs an agreement-oriented response from element-wise multiplication and a discrepancy-oriented response from absolute difference. An adaptive selector estimates their relative contributions at each spatial-channel location, after which the selected enhancement is injected into a direct concatenation-based fusion path through a learnable residual scale. CCE therefore performs response-type selection after interaction rather than another round of modality-level competition.

Taken together, LB-CIM differs from existing multimodal fusion designs not simply by combining denoising, attention, and gating operations, but by assigning these mechanisms distinct roles along the information flow. MCD improves SAR reliability before interaction, DLBCA restricts interaction bandwidth and selectively aggregates differential information during interaction, and CCE reorganizes agreement- and discrepancy-oriented responses after interaction. These design choices are further examined through targeted ablation and mechanism-replacement experiments in Section 3.4.

The main contributions of this work are summarized as follows:

- We propose LB-CIM, a progressive VIS–SAR fusion module that organizes cross-modal learning into three consecutive stages: SAR reliability refinement, low-bandwidth differential interaction, and consensus-complementary enhancement. The module can be incorporated at multiple feature scales of a dual-branch detector while preserving the basic detection pipeline.
- We design MCD to characterize potentially unreliable SAR responses using local residual and cross-scale consistency cues. Instead of globally filtering SAR features, MCD estimates an element-wise suppression weight and applies it to a signed residual, enabling selective attenuation of unstable responses while retaining informative structural cues.
- We introduce DLBCA to control both the bandwidth and content of bidirectional cross-modal interaction. Queries retain the unreduced spatial resolution of the compact interaction grid, whereas channel-compressed and further spatially reduced key/value representations limit the amount of information involved in cross-modal matching. Differential value tokens determine the information transferred between modalities, and a local difference-aware gate regulates the resulting residual updates.
- We develop CCE to perform response-type selection after bidirectional interaction. Element-wise multiplication and absolute difference are used to construct agreement-oriented and discrepancy-oriented descriptors, respectively, and an adaptive selector regulates their relative contributions. The selected enhancement is then injected into a direct fusion path through a learnable residual scale.

Extensive comparative experiments and ablation studies are conducted to evaluate the individual contribution of each component and the combined effect of the progressive fusion strategy on VIS–SAR object detection.

## 2. Materials and Methods

### 2.1. Overall Framework

**Figure 1:**
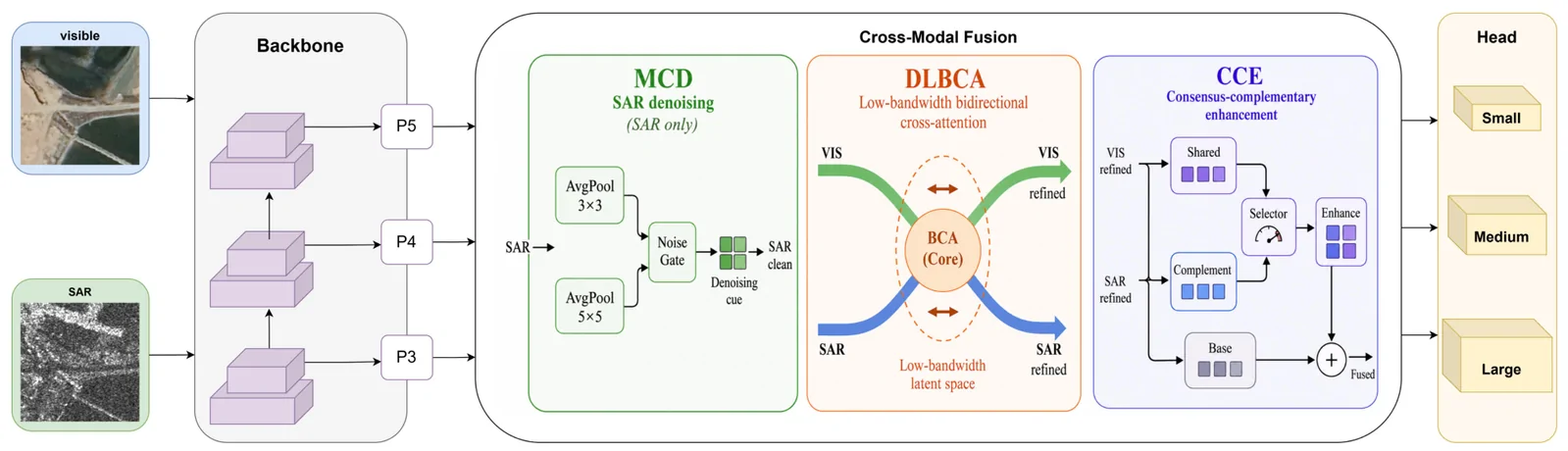
Overall framework of the proposed VIS–SAR object detector. The visible and SAR branches extract hierarchical features at P3, P4, and P5. At each feature level, LB-CIM performs progressive SAR-specific denoising, bidirectional cross-modal interaction, and consensus-complementary enhancement. The fused features are then integrated by the standard YOLO11 multi-scale neck and delivered to a single detection head for multi-scale object prediction.

The overall architecture of the proposed VIS–SAR object detector is illustrated in Figure 1. The implementation follows a dual-branch YOLO11n architecture. Each paired sample is organized as a four-channel input containing a three-channel visible image and a single-channel SAR image. The two modalities are separated before feature extraction and processed by structurally identical VIS and SAR backbone branches. Although the two branches use the same sequence of convolutional and C3k2 stages, their backbone parameters are independently learned rather than shared. This design preserves modality-specific feature extraction while maintaining matched feature resolutions and channel dimensions for subsequent cross-modal interaction.

For the YOLO11n configuration used in this study, the two branches generate matched feature pairs at three pyramid levels. Given an input resolution of 640×640, each branch produces a P3 feature with dimensions of 128×80×80, a P4 feature with dimensions of 128×40×40, and a P5 feature with dimensions of 256×20×20. These levels correspond to spatial strides of 8, 16, and 32, respectively. At each level, LB-CIM receives the paired VIS and SAR features, with input channel configurations of 128+128, 128+128, and 256+256 for P3, P4, and P5, respectively. It produces a fused representation with the same channel and spatial dimensions as either input branch. The detailed configuration is summarized in Table 1.

The two backbone branches produce hierarchical feature pairs $\left\{F_{v}^{l},F_{s}^{l}\right\}$, where l∈{3,4,5} denotes the P3, P4, and P5 levels, respectively. The higher-resolution P3 representation retains relatively rich spatial details, whereas P4 and P5 progressively encode higher-level semantic information at lower spatial resolutions. This hierarchical organization follows the general principle of feature pyramids, which exploit representations at different semantic and spatial scales to improve object detection across scale variations [34]. Such multi-scale processing is particularly relevant to remote sensing imagery [35], where objects can exhibit substantial variations in spatial extent and many targets occupy only a limited number of pixels [36]. In the complete LB-CIM configuration used in the main experiments, LB-CIM is applied at P3, P4, and P5 so that cross-modal interaction can be performed across multiple pyramid levels. This three-level placement is treated as an empirical design choice rather than an a priori optimal setting, and the contributions of individual and combined fusion levels are evaluated in Section 3.4.5.

At each feature level, LB-CIM follows a progressive reliability–interaction–enhancement procedure. Given the original VIS and SAR features $F_{v}^{l}$ and $F_{s}^{l}$, MCD first refines the SAR feature while preserving its spatial resolution. The VIS feature and the MCD-refined SAR feature are separately processed by adaptive average pooling and adaptive max pooling to obtain the prescribed interaction-grid sizes. For each modality, the two pooled representations are then combined using modality-specific learnable softmax-normalized weights. DLBCA performs bidirectional differential interaction on these compact features, and CCE subsequently consolidates their consensus and complementary responses. To obtain the final pyramid representation, the CCE output is restored to the original spatial resolution and combined with a separate full-resolution fusion path formed from the original VIS and SAR features. The overall processing is summarized in Equation (1).

(1) $$\begin{array}{cc} F_{s}^{d,l} & =\mathrm{MCD}(F_{s}^{l}),\\ \left(F_{v}^{\prime ,l},F_{s}^{\prime ,l}\right) & =\mathrm{DLBCA}\left(P_{v}^{l}\left(F_{v}^{l}\right),P_{s}^{l}\left(F_{s}^{d,l}\right)\right),\\ F_{\mathrm{ctx}}^{l} & =\mathrm{CCE}(F_{v}^{\prime ,l},F_{s}^{\prime ,l}),\\ F_{\mathrm{out}}^{l} & =\phi_{\mathrm{base}}^{l}\left(\left[F_{v}^{l};F_{s}^{l}\right]\right)+\gamma^{l}⊙\phi_{\mathrm{proj}}^{l}\left(U_{H_{l},W_{l}}\left(F_{\mathrm{ctx}}^{l}\right)\right). \end{array}$$

where $P_{v}^{l}$ and $P_{s}^{l}$ denote the modality-specific learnable combinations of adaptive average and max pooling. $U_{H_{l},W_{l}}$ represents bilinear interpolation to the original spatial resolution, and $\phi_{\mathrm{proj}}^{l}$ denotes an optional channel projection. The full-resolution base fusion $\phi_{\mathrm{base}}^{l}$ is implemented using a 1×1 Conv-BN-SiLU block, where $\gamma^{l}$ is a learnable channel-wise residual scaling parameter initialized to $10^{-3}$.

For clarity, the pyramid-level superscript *l* is omitted in the following module descriptions; thus, $F_{s}^{d}$, $F_{v}^{\prime }$, $F_{s}^{\prime }$, $F_{\mathrm{ctx}}$, and $F_{\mathrm{out}}$ refer to the corresponding features at the pyramid level under discussion.

In the complete LB-CIM configuration, the three stages are arranged as MCD → DLBCA → CCE. The motivation for placing MCD before cross-modal interaction is to refine potentially unreliable SAR responses before they participate in bidirectional information exchange. This ordering is motivated by the intended reliability–interaction–enhancement information flow rather than being assumed to be intrinsically optimal, and its effect is explicitly evaluated in Section 3.4.5. DLBCA subsequently performs bidirectional interaction between the compact representations derived from $F_{v}^{l}$ and $F_{s}^{d,l}$, using differential information transfer. The resulting $F_{v}^{\prime ,l}$ and $F_{s}^{\prime ,l}$ retain separate modality streams while incorporating information from the opposite branch. CCE then consolidates these interacted representations by adaptively balancing consensus-oriented and complementary-oriented responses. Accordingly, the three stages perform SAR reliability refinement, selective cross-modal interaction, and post-interaction feature consolidation in sequence. Their detailed implementations are described in Section 2.2, Section 2.3 and Section 2.4.

The fused P5 feature is first processed by the SPPF and C2PSA blocks. The resulting feature then enters the standard YOLO11 multi-scale neck. In the top-down pathway, it is upsampled and concatenated with the fused P4 feature, followed by a C3k2 block. The resulting feature is further upsampled and concatenated with the fused P3 feature, followed by another C3k2 block. In the bottom-up pathway, stride-2 convolutions and feature concatenation are used to reconstruct the P4- and P5-level neck representations, with C3k2 blocks applied after the corresponding fusion operations. Finally, the three neck outputs at P3, P4, and P5 are delivered to a single standard YOLO11 detection head for multi-scale object classification and localization. Apart from introducing LB-CIM as the fusion operator at P3, P4, and P5, the remaining neck and detection-head configuration is kept unchanged.

### 2.2. Multi-Scale Consistency Denoising

#### 2.2.1. Motivation and Overview

SAR imagery provides structural and scattering information that is complementary to visible observations [37]. However, its coherent imaging mechanism also gives rise to speckle and locally unstable responses. This issue becomes more relevant in feature-level multimodal fusion, because unreliable SAR activations may be propagated to the visible branch once cross-modal interaction is performed. A variety of deep-learning-based SAR despeckling strategies have been developed, including supervised, residual, self-supervised, and noise-referenced learning approaches [38,39,40,41,42,43]. A straightforward solution is to smooth the SAR features before fusion. Such uniform filtering, however, cannot distinguish undesirable fluctuations from informative high-frequency responses associated with target boundaries or scattering structures.

**Figure 2:**
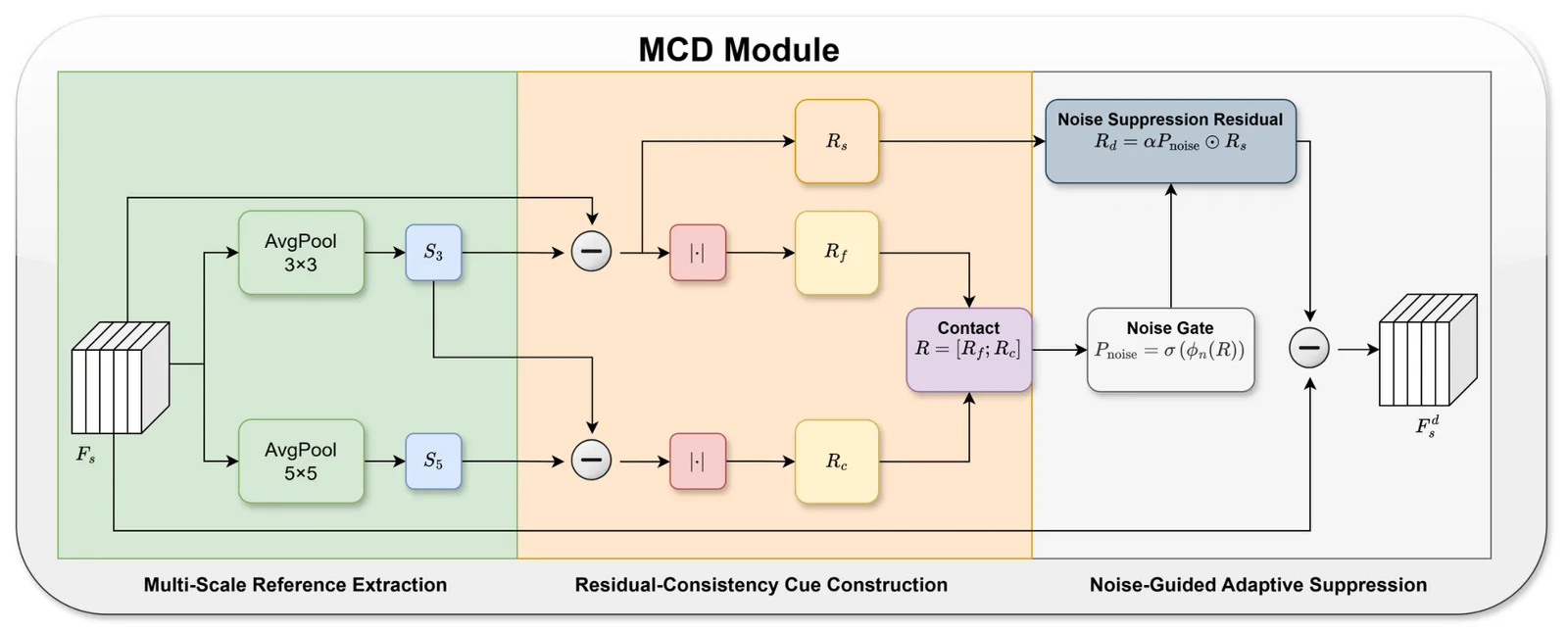
Detailed structure of the proposed Multi-Scale Consistency Denoising (MCD) module. Multi-scale local references are first constructed from the SAR feature. Local residual and cross-scale discrepancy cues are then used to estimate a noise-aware suppression response, which modulates the signed residual before residual subtraction.

The proposed Multi-Scale Consistency Denoising (MCD) module is designed from this perspective. Instead of treating all local high-frequency responses as noise, MCD evaluates their deviation from local references and their variation across different smoothing scales. These cues are used to estimate an adaptive suppression response, which subsequently modulates a signed local residual. As illustrated in Figure 2, MCD consists of three successive stages: multi-scale reference extraction, residual-consistency cue construction, and noise-guided adaptive suppression. Given an input SAR feature $F_{s}\in R^{C\times H\times W}$, the module outputs a refined representation $F_{s}^{d}\in R^{C\times H\times W}$ with unchanged spatial and channel dimensions.

#### 2.2.2. Multi-Scale Reference Extraction

The first stage constructs two local reference features at different spatial scales. Specifically, the input SAR feature $F_{s}$ is processed using 3×3 and 5×5 average pooling:

(2) $$\begin{array}{cc} S_{3} & ={\mathrm{AvgPool}}_{3\times 3}\left(F_{s}\right),\\ S_{5} & ={\mathrm{AvgPool}}_{5\times 5}\left(F_{s}\right), \end{array}$$

where $F_{s},S_{3},S_{5}\in R^{C\times H\times W}$, and $S_{3}$ and $S_{5}$ denote the local reference features obtained at the two scales, respectively. The pooling operations preserve the spatial dimensions of the input feature. The smaller 3×3 neighborhood retains relatively fine local variations and therefore provides the primary reference for residual construction, whereas the larger 5×5 neighborhood supplies a broader reference for evaluating the stability of local responses across scales.

Neither pooled representation is directly treated as a denoised feature. Instead, their relationships with the original SAR feature and with each other are exploited in the subsequent residual-consistency analysis. This allows MCD to characterize local response variation without imposing uniform smoothing on the complete feature map.

#### 2.2.3. Residual-Consistency Cue Construction

Based on the multi-scale references, MCD constructs a signed residual together with two unsigned cues:

(3) $$\begin{array}{cc} R_{s} & =F_{s}-S_{3},\\ R_{f} & =|F_{s}-S_{3}|,\\ R_{c} & =|S_{3}-S_{5}|,\\ R & =[R_{f};R_{c}]. \end{array}$$

Here, |·| denotes element-wise absolute value and [·;·] represents channel-wise concatenation. The signed residual $R_{s}\in R^{C\times H\times W}$ preserves the direction of the local deviation from $S_{3}$ and is retained for subsequent feature correction. In contrast, the fine residual cue $R_{f}$ characterizes only the magnitude of this deviation and is used for noise-aware estimation.

The cross-scale cue $R_{c}$ measures the discrepancy between the two local reference features. Rather than being regarded as a deterministic noise indicator, it provides an auxiliary description of the sensitivity of local responses to a change in spatial scale. Together, $R_{f}$ and $R_{c}$ form $R\in R^{2C\times H\times W}$, which is subsequently used to estimate the suppression response. In this way, MCD separates the cues used to determine where suppression is required from the signed residual that is eventually modified.

#### 2.2.4. Noise-Guided Adaptive Suppression

The aggregated cue *R* is passed through a lightweight gating function to generate an adaptive suppression response:

(4) $$P_{\mathrm{noise}}=\sigma \left(\phi_{n}\left(R\right)\right),$$

where $\phi_{n}\left(\cdot \right)$ denotes the lightweight feature transformation in the noise gate and σ(·) is the sigmoid function. The resulting $P_{\mathrm{noise}}\in {\left[0,1\right]}^{C\times H\times W}$ provides element-wise modulation of the signed residual. Although denoted as $P_{\mathrm{noise}}$ for convenience, it is used as a learned noise-aware gating response rather than a calibrated statistical probability.

MCD further introduces a learnable coefficient to regulate the overall suppression strength. Let $\theta_{\alpha }$ denote the underlying learnable parameter. The effective coefficient is defined as

(5) $$\alpha =\sigma (\theta_{\alpha }),$$

which constrains α to the interval (0,1).

The residual component to be removed is then calculated as

(6) $$R_{d}=\alpha P_{\mathrm{noise}}⊙R_{s},$$

where ⊙ denotes element-wise multiplication and $R_{d}$ represents the noise suppression residual. In this formulation, $P_{\mathrm{noise}}$ determines the spatial-channel distribution of the correction, α controls its overall magnitude, and $R_{s}$ preserves the signed direction of the local deviation.

Finally, the refined SAR representation is obtained by subtracting the modulated residual from the original feature:

(7) $$F_{s}^{d}=F_{s}-R_{d}.$$

Substituting $R_{s}=F_{s}-S_{3}$ into Equation (6) and then into Equation (7) yields the complete MCD formulation:

(8) $$F_{s}^{d}=F_{s}-\alpha P_{\mathrm{noise}}⊙\left(F_{s}-S_{3}\right).$$

This formulation performs suppression only on the gated residual rather than directly modifying the complete SAR representation. Responses assigned a weak gating value remain close to their original form, whereas locally unstable components receiving stronger responses undergo a larger correction. Consequently, MCD provides a selective preprocessing stage for the SAR branch while retaining the structural information required for subsequent cross-modal interaction. The resulting $F_{s}^{d}$ is then paired with the visible feature $F_{v}$ and passed to DLBCA.

### 2.3. Differential Low-Bandwidth Cross-Attention

#### 2.3.1. Motivation and Overview

After MCD refinement, the SAR feature $F_{s}^{d}$ is paired with the corresponding visible feature $F_{v}$ for cross-modal interaction. Cross-attention provides an effective means of establishing long-range dependencies between heterogeneous modalities. In conventional cross-attention, however, the complete value representation of one modality is directly transferred to the other according to the learned query–key correspondence. For VIS–SAR features with markedly different imaging characteristics, such dense exchange may propagate information that is already shared by the two modalities together with redundant or inconsistent responses. Moreover, repeatedly applying dense cross-attention over unreduced pyramid features at multiple feature levels increases the amount of cross-modal token interaction.

**Figure 3:**
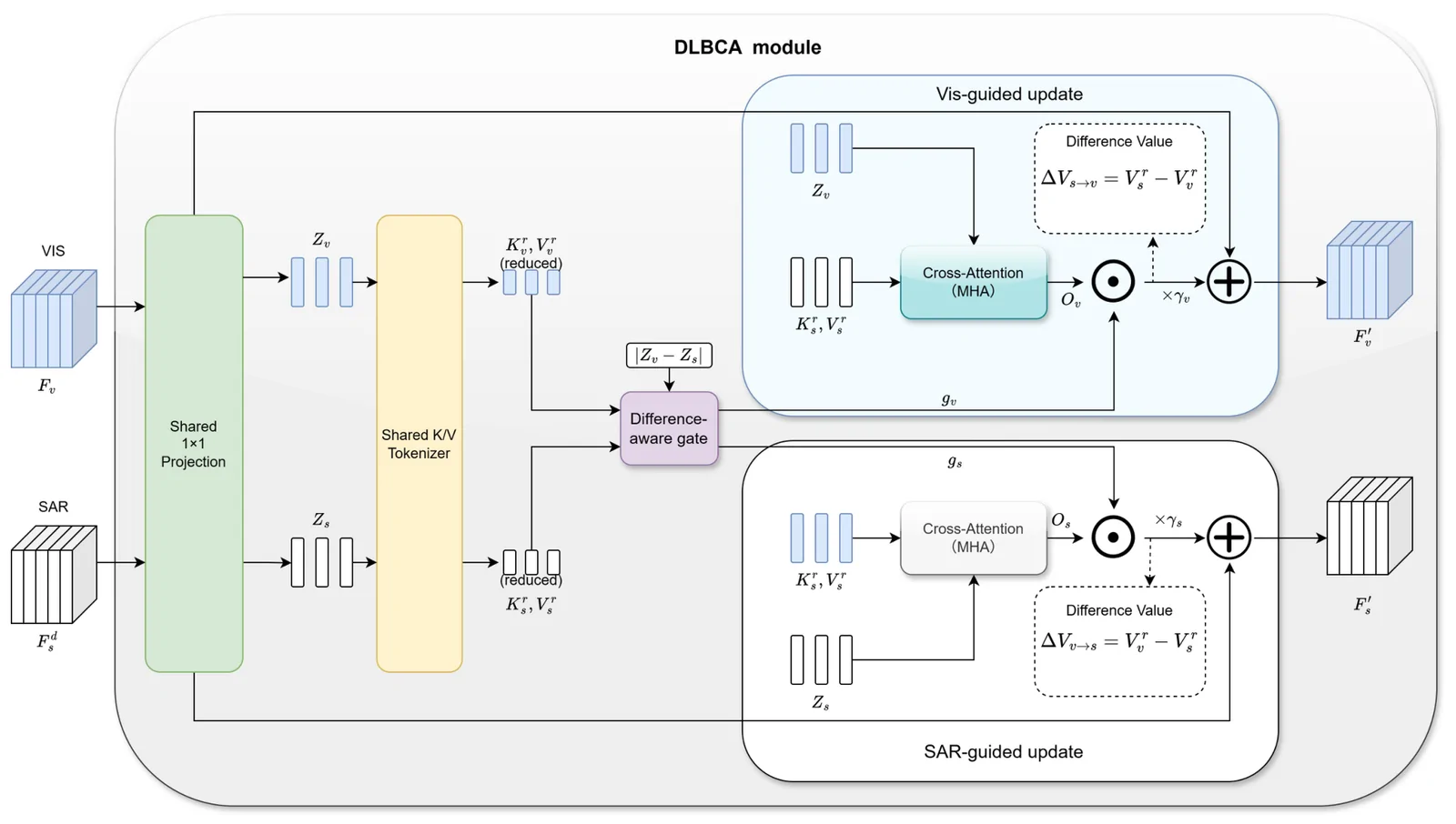
Detailed structure of the proposed Differential Low-Bandwidth Cross-Attention (DLBCA) module. Queries retain the full resolution of the compact interaction grid, while keys and values are further spatially reduced. Bidirectional attention aggregates differential values, which are regulated by a difference-aware gate before residual updating. The positional encoding and spatial-gated FFN are described in Section 2.3.3 but omitted here for clarity.

To make the interaction more selective, we design Differential Low-Bandwidth Cross-Attention (DLBCA), as illustrated in Figure 3. The module reduces the information involved in cross-modal interaction in two ways. First, the modality features are projected into a compact latent space, while only the key and value sequences are spatially reduced; the queries retain their original spatial resolution. Second, instead of transferring the complete value representation of the opposite modality, DLBCA exchanges directional differences between the two value representations. A local difference-aware gate is further introduced to regulate the transferred response before residual updating. The same procedure is performed in both VIS-guided and SAR-guided directions, producing the refined features $F_{v}^{\prime }$ and $F_{s}^{\prime }$ for subsequent consensus–complementary fusion.

#### 2.3.2. Compact Interaction and Differential-Value Aggregation

Before entering DLBCA, the paired VIS and SAR features at each pyramid level are mapped to a compact interaction grid. For the 640 × 640 input setting, the P3 and P4 features are mapped to 16 × 16 grids, whereas the P5 features are mapped to an 8 × 8 grid. This mapping is implemented using a custom target-size pooling operation. Specifically, the average- and max-pooling branches determine their kernel sizes and strides according to the input and target grid dimensions. Their outputs are then combined using modality-specific learnable normalized weights. This spatial compression limits the resolution involved in cross-modal interaction, while the original-resolution VIS and SAR features are retained by the parallel base fusion path described in Section 2.1. For clarity, the resulting compact VIS and SAR features are denoted by $F_{v}$ and $F_{s}^{d}$, respectively, in the following  formulation.

Given $F_{v},F_{s}^{d}\in R^{B\times C\times H_{a}\times W_{a}}$, a shared 1×1 projection first maps the two modalities into an attention space with channel dimension $C_{a}$. Queries are generated directly from the projected features, whereas keys and values are obtained from a shared spatial tokenizer. The overall transformation is written as

(9) $$\begin{array}{cccc} Z_{v} & =P(F_{v}), & Z_{s} & =P(F_{s}^{d}),\\ Q_{v} & =W_{q}Z_{v}, & Q_{s} & =W_{q}Z_{s},\\ T_{v} & =T(Z_{v}), & T_{s} & =T(Z_{s}),\\ \left(K_{v}^{r},V_{v}^{r}\right) & =(W_{k}T_{v},W_{v}T_{v}), & \left(K_{s}^{r},V_{s}^{r}\right) & =(W_{k}T_{s},W_{v}T_{s}), \end{array}$$

where P(·) denotes the shared 1×1 channel projection, and $W_{q}$, $W_{k}$, and $W_{v}$ are the query, key, and value projections shared by the two modalities. The attention channel dimension is set to $C_{a}=0.5C$, and four attention heads are used at all three pyramid levels. Accordingly, the dimensionality of each head is $d=C_{a}/4$.

The spatial tokenizer T(·) is shared by the VIS and SAR branches and consists of a 2×2 average-pooling operation with stride 2, followed by a depthwise 3×3 convolution and a SiLU activation. Queries are generated directly from the projected compact features, whereas keys and values are generated from the further reduced tokenizer outputs.

Let $N=H_{a}W_{a}$ denote the number of query tokens and $M=\left(H_{a}/2\right)\left(W_{a}/2\right)$ denote the number of reduced key/value tokens. After flattening the spatial dimensions and splitting the channels into multiple heads, the tensor dimensions are

$$Q_{v},Q_{s}\in R^{B\times h\times N\times d},$$

$$K_{v}^{r},K_{s}^{r}\in R^{B\times h\times M\times d},\;V_{v}^{r},V_{s}^{r}\in R^{B\times h\times M\times d}.$$

where h=4 and $d=C_{a}/h$. Thus, the P3 and P4 levels contain 256 query tokens and 64 key/value tokens, whereas P5 contains 64 query tokens and 16 key/value tokens. The detailed configurations used at the three pyramid levels are summarized in Table 2.

The above configuration is used as the default setting of the complete model. The attention channel ratio of 0.5 reduces the latent channel dimension while retaining sufficient representation capacity for cross-modal matching, while four heads are used to provide multiple interaction subspaces with limited additional complexity. A spatial reduction factor of r=2 further decreases the number of key/value tokens while avoiding overly aggressive spatial compression. These values are treated as practical accuracy–complexity settings rather than assumed optima; the effect of the spatial reduction factor is examined separately in the sensitivity experiments.

For reproducibility, the initial spatial compression is implemented using a custom fixed-window pooling routine. Although the implementation helper is named AdaptivePool2d, it does not use the standard adaptive average- or max-pooling operators. Instead, it calculates the pooling kernel size and stride from the input and target grid dimensions, and applies F.avg_pool2d or F.max_pool2d without padding. For an input resolution of 640×640, the P3 mapping from 80×80 to 16×16 uses a 5×5 kernel with stride 5; the P4 mapping from 40×40 to 16×16 uses a 10×10 kernel with stride 2; and the P5 mapping from 20×20 to 8×8 uses a 6×6 kernel with stride 2. The P4 and P5 pooling windows therefore overlap, whereas the P3 windows do not. The average- and max-pooled outputs are subsequently combined using separate modality-specific learnable weights normalized by softmax. This initial compression is distinct from the subsequent shared DLBCA tokenizer, which applies a fixed 2×2 average-pooling operation with stride 2, as described above.

The central operation of DLBCA is attention-weighted differential-value aggregation. Because the paired VIS and SAR inputs are spatially aligned and the same spatial tokenizer is applied to both branches, the reduced value tokens at the same index correspond to approximately the same nominal spatial region. DLBCA therefore first constructs directional differential-value sequences on the shared reduced grid:

$$\Delta V_{s\to v}^{r}=V_{s}^{r}-V_{v}^{r},\;\Delta V_{v\to s}^{r}=V_{v}^{r}-V_{s}^{r}.$$

For the VIS-guided direction, cross-modal correspondence is estimated between the visible queries and the reduced SAR keys, whereas the reverse direction uses the SAR queries and the reduced visible keys. The bidirectional interaction is expressed as

(10) $$\begin{array}{cc} O_{v} & =\mathrm{Softmax}\left(\frac{Q_{v}{\left(K_{s}^{r}\right)}^{T}}{\sqrt{d}}\right)\left(V_{s}^{r}-V_{v}^{r}\right),\\ O_{s} & =\mathrm{Softmax}\left(\frac{Q_{s}{\left(K_{v}^{r}\right)}^{T}}{\sqrt{d}}\right)\left(V_{v}^{r}-V_{s}^{r}\right), \end{array}$$

where *d* is the dimensionality of each attention head. In each direction, the attention matrix has dimensions B×h×N×M, and the attention-weighted differential-value aggregation produces an output of size B×h×N×d. After concatenating all heads and reshaping, the directional output has dimensions $R^{B\times C_{a}\times H_{a}\times W_{a}}$. The subsequent difference-aware gating and shared output projection map the cross-modal updates back to the original compact feature dimension *C*, yielding $F_{v}^{\prime }$ and $F_{s}^{\prime }$ with dimensions $R^{B\times C\times H_{a}\times W_{a}}$. Therefore, the DLBCA outputs retain the compact interaction-grid resolution. Restoration to the original pyramid resolution is performed after CCE through bilinear interpolation, as described in Section 2.1.

To clarify the operation in Equation (10), let

$$A_{v}=\mathrm{Softmax}\left(\frac{Q_{v}{\left(K_{s}^{r}\right)}^{T}}{\sqrt{d}}\right).$$

For a visible query at position *i*, the corresponding update can be written as

$$O_{v,i}=\sum_{j}A_{ij}^{v}\left(V_{s,j}^{r}-V_{v,j}^{r}\right).$$

Thus, the subtraction is performed between co-indexed VIS and SAR value tokens on the reduced spatial grid before attention aggregation. The attention weights do not independently retrieve a SAR value and then subtract a visible value at the receiving query position. Instead, $A_{v}$ acts as a cross-modal correspondence distribution over a pre-constructed differential-value field. Equivalently,

$$O_{v}=A_{v}V_{s}^{r}-A_{v}V_{v}^{r}.$$

The second term should therefore not be interpreted as a separate VIS self-attention operation; it represents the VIS component of the same spatially paired differential tokens selected according to VIS-to-SAR correspondence.

This formulation differs from conventional cross-attention primarily in the information carried by the value sequence. Standard cross-attention would transfer

$$O_{v}^{\mathrm{CA}}=A_{v}V_{s}^{r},$$

so that the complete counterpart value representation participates in the update. In DLBCA, the transferred sequence is instead replaced by $\Delta V_{s\to v}^{r}$. Consequently, responses that are similar in the two modalities are attenuated before aggregation, whereas discrepancies between the paired representations remain available for selective transfer. This operation also differs from computing a local difference only after standard cross-attention. In the latter case, the counterpart representation is first aggregated and the discrepancy is subsequently evaluated at the receiving location. DLBCA instead constructs the cross-modal difference on the reduced, spatially paired token grid before aggregation and then uses the cross-modal correspondence to determine which differential tokens contribute to each query.

The differential construction relies on approximate spatial correspondence between the paired VIS and SAR representations. In the datasets considered in this work, the two modalities are spatially registered, and identical pooling and tokenization operations are applied to both branches. Therefore, the *j*-th reduced VIS and SAR tokens refer to the same nominal spatial region. Residual registration errors may nevertheless introduce non-semantic differences, which motivates the subsequent difference-aware gating mechanism. The effects of standard value transfer, differential-value transfer, and alternative differencing strategies are further examined in the mechanism-level ablation experiments.

#### 2.3.3. Difference-Aware Gated Updating

The differential attention described above captures cross-modal relationships over the reduced key/value sequences. To further regulate the transferred information, DLBCA derives a local difference representation from the projected compact features $Z_{v}$ and $Z_{s}$, which are aligned in spatial resolution. This representation is used to generate modality-specific gates for the subsequent differential updates:

(11) $$\begin{array}{cc} D & =\phi_{d}\left(|Z_{v}-Z_{s}|\right),\\ G_{v} & =\sigma \left(\phi_{g}\left(I(Z_{v},D)\right)\right),\\ G_{s} & =\sigma \left(\phi_{g}\left(I(Z_{s},D)\right)\right), \end{array}$$

where |·| denotes the element-wise absolute difference, $\phi_{d}\left(\cdot \right)$ is implemented using a depthwise 3×3 convolution to refine the local discrepancy representation, and I(·,·) denotes the channel-wise paired interleaving operation. The shared gating transformation $\phi_{g}\left(\cdot \right)$ consists of a grouped 1×1 convolution, a SiLU activation, and a depthwise 3×3 convolution, followed by a sigmoid function. The same gating parameters are shared by the two modalities, while their inputs are conditioned on the respective modality features, producing separate gating maps $G_{v},G_{s}\in {\left[0,1\right]}^{B\times C_{a}\times H_{a}\times W_{a}}$. These gates independently regulate the amount of differential information incorporated into the visible and SAR branches, allowing the model to selectively use cross-modal discrepancies rather than directly injecting all differential responses.

The attention responses are first modulated by the corresponding gates and then projected back to the original feature dimension. With learnable residual scales $\gamma_{v}$ and $\gamma_{s}$, the final updates are written as

(12) $$\begin{array}{cc} F_{v}^{\prime } & =F_{v}+\gamma_{v}W_{o}\left(G_{v}⊙O_{v}\right),\\ F_{s}^{\prime } & =F_{s}^{d}+\gamma_{s}W_{o}\left(G_{s}⊙O_{s}\right), \end{array}$$

where $W_{o}$ denotes the shared 1×1 output projection, which maps the attention dimension $C_{a}$ back to the original feature dimension *C*, and ⊙ denotes element-wise multiplication. The learnable channel-wise residual scales $\gamma_{v}$ and $\gamma_{s}$ are independently maintained for the two modalities and initialized to 0.01. These small initial values allow the model to gradually incorporate cross-modal corrections while preserving the original modality-specific representations.

In the implementation, the differential cross-attention core is embedded in a lightweight transformer refinement block. Before cross-modal interaction, a shared depthwise 3×3 convolution is applied to each compact modality feature through a residual connection to provide convolutional positional encoding. Both the convolutional weights and biases are initialized to zero. Separate LayerNorm2d operations are then applied to the two modality features before cross-modal attention.

After the attention-based residual updates, a shared spatial-gated feed-forward network further refines the two modality representations. It consists of a 1×1 channel expansion, a depthwise 3×3 convolution, channel splitting, SiLU-based multiplicative gating, and a final 1×1 projection. The expansion ratio is set to 1.0. Separate normalization layers and learnable channel-wise residual scales are maintained for the two modality branches, with the residual scales initialized to 0.01. These auxiliary operations complement the differential cross-modal interaction and are omitted from Figure 3 for clarity.

The resulting interaction follows a complementary division of roles. Spatially reduced K/V representations control the amount of information involved in cross-modal matching; directional value differences determine what information is transferred; and the local difference-aware gates regulate how strongly this information modifies each modality. The refined features $F_{v}^{\prime }$ and $F_{s}^{\prime }$ preserve the two modality streams and are subsequently forwarded to CCE for final consensus–complementary fusion.

### 2.4. Consensus-Complementary Enhancement

#### 2.4.1. Motivation and Overview

DLBCA enables bidirectional differential interaction between the visible and SAR branches, producing the refined modality features $F_{v}^{\prime }$ and $F_{s}^{\prime }$. Although both features have incorporated information from the opposite modality, they remain two separate representations in which cross-modal agreement and modality-dependent differences are still mixed. Direct concatenation or modality-wise weighting can combine these features, but such operations do not explicitly distinguish information jointly supported by both modalities from information arising from their complementary responses.

**Figure 4:**
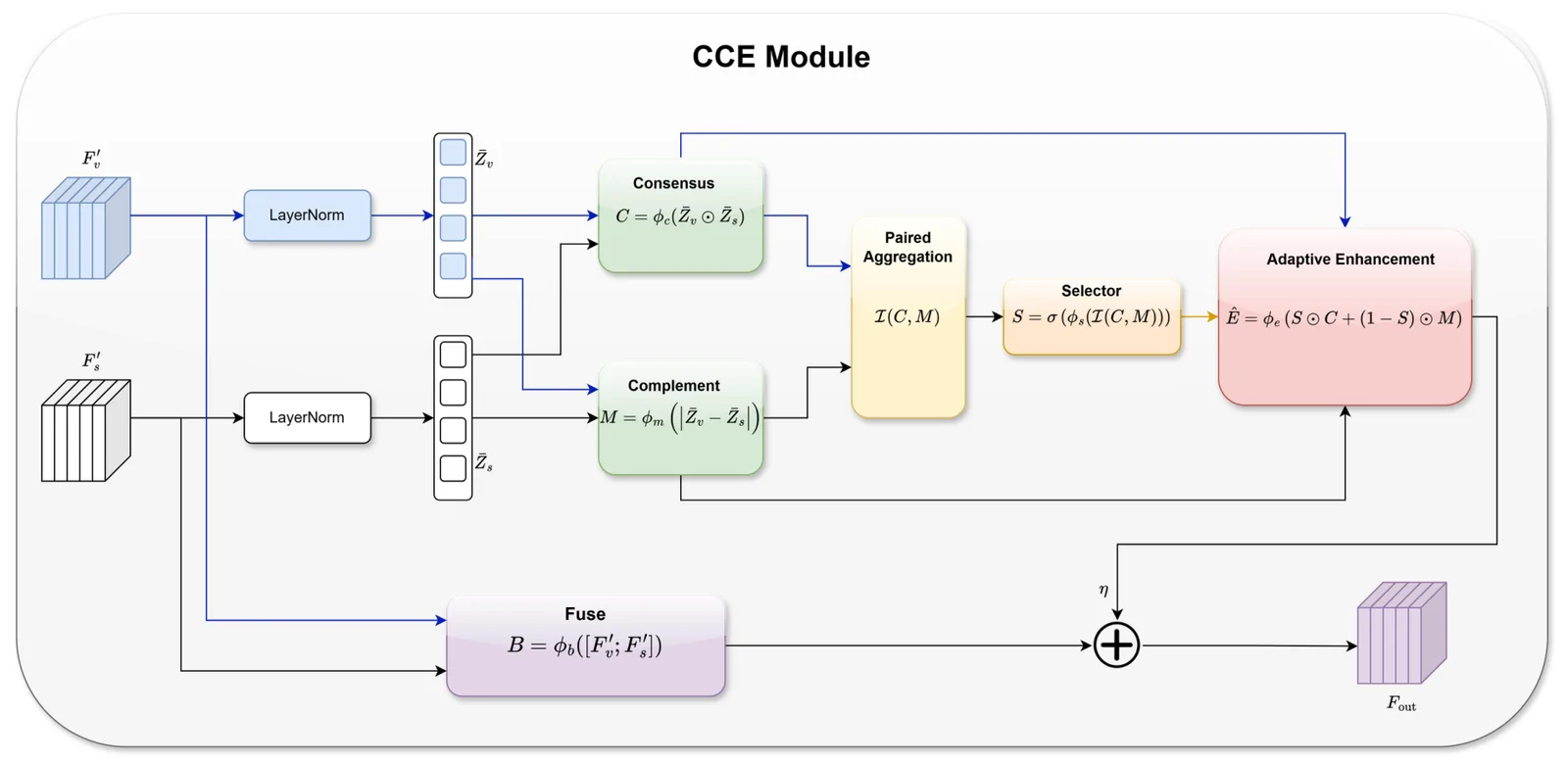
Detailed structure of the proposed Consensus-Complementary Enhancement (CCE) module. The DLBCA-refined visible and SAR features are normalized and used to construct consensus-oriented and complementary-oriented responses. An adaptive selector balances the two response types, and the resulting enhancement is projected and added to a base fusion representation through a learnable residual scale.

To further organize the interacted representations, we introduce the Consensus-Complementary Enhancement (CCE) module, as illustrated in Figure 4. Instead of assigning a single relative importance to the visible and SAR modalities, CCE models two different types of cross-modal evidence. A product-based branch encodes the element-wise polarity relationships between the normalized modality features, while a difference-based branch characterizes the magnitude of their local discrepancies. An adaptive selector then regulates the relative contribution of these two descriptors. The selected enhancement is finally injected into a base fusion representation through a learnable residual scale. In this way, CCE performs information-type selection after cross-modal interaction rather than another round of modality-level competition.

#### 2.4.2. Consensus-Complementary Representation

Given the DLBCA outputs $F_{v}^{\prime },F_{s}^{\prime }\in R^{C\times H\times W}$, modality-specific layer normalization is first applied to reduce the influence of feature-scale differences:

(13) $$\begin{array}{cc} {\bar{Z}}_{v} & =LN_{v}\left(F_{v}^{\prime }\right),{\bar{Z}}_{s}=LN_{s}\left(F_{s}^{\prime }\right),\\ P & ={\bar{Z}}_{v}⊙{\bar{Z}}_{s},D=\left|{\bar{Z}}_{v}-{\bar{Z}}_{s}\right|,\\ C & =\phi_{c}\left(P\right),M=\phi_{m}\left(D\right). \end{array}$$

where $LN_{v}\left(\cdot \right)$ and $LN_{s}\left(\cdot \right)$ denote the normalization operations for the visible and SAR branches, respectively. *P* and *D* represent the element-wise product and absolute difference of the normalized features, respectively. The functions $\phi_{c}\left(\cdot \right)$ and $\phi_{m}\left(\cdot \right)$ are lightweight refinement transformations implemented using depthwise 3×3 convolution followed by SiLU activation.

The product descriptor *P* encodes the element-wise polarity relationship between the two modalities. Since layer-normalized features contain both positive and negative responses, a positive product indicates same-polarity responses, including both positive-positive and negative-negative patterns, whereas a negative product indicates opposite-polarity responses. Therefore, *P* should not be interpreted as a direct indicator of target presence or guaranteed semantic agreement. Instead, it provides a polarity-aware cross-modal interaction descriptor, which is further refined by the subsequent convolution and nonlinear activation in $\phi_{c}\left(\cdot \right)$.

In contrast, the absolute difference descriptor *D* captures the magnitude of cross-modal discrepancies without explicitly preserving their direction. It indicates the extent of response differences but does not distinguish which modality exhibits a stronger response. The adaptive selector subsequently regulates the relative contributions of *C* and *M* according to their local feature context, rather than explicitly identifying the modality responsible for a particular discrepancy. These two descriptors provide complementary information for subsequent adaptive fusion, rather than an exact mathematical decomposition of common and modality-specific information.

#### 2.4.3. Adaptive Selection and Residual Enhancement

The product-based and difference-based descriptors are jointly used to estimate an adaptive selection map. The selection and enhancement process can be expressed as

(14) $$\begin{array}{cc} S & =\sigma \left(\phi_{s}\left(I(C,M)\right)\right),\\ \hat{E} & =\phi_{e}\left(S⊙C+(1-S)⊙M\right), \end{array}$$

where I(C,M) denotes the paired channel aggregation of the two descriptors, $\phi_{s}\left(\cdot \right)$ represents the lightweight selector, and σ(·) is the sigmoid function. Accordingly, $S\in {\left[0,1\right]}^{C\times H\times W}$, and $\phi_{e}\left(\cdot \right)$ denotes a 1×1 projection used to obtain the final enhancement feature $\hat{E}$.

The selector *S* determines the relative contribution of the two descriptors at each spatial-channel location. A larger value of *S* places greater emphasis on the product-based descriptor *C*, whereas a smaller value increases the contribution of the difference-based descriptor *M*. Therefore, CCE does not impose a fixed preference for either descriptor. Instead, their contributions are adaptively adjusted according to the local feature context.

In parallel with the enhancement branch, the original DLBCA outputs are preserved through a base fusion path. The final fusion process is written as

(15) $$\begin{array}{cc} B & =\phi_{b}\left([F_{v}^{\prime };F_{s}^{\prime }]\right),\\ F_{\mathrm{out}} & =B+\eta \hat{E}, \end{array}$$

where [·;·] denotes channel-wise concatenation, $\phi_{b}\left(\cdot \right)$ represents the base fusion transformation consisting of concatenation followed by a 1×1 convolution, and $B\in R^{C\times H\times W}$ is the baseline fused representation. The parameter η is a learnable scaling factor initialized with a small value to regulate the contribution of the enhancement branch.

The base path preserves the direct joint representation of the two refined modalities, while the enhancement branch introduces additional polarity-aware and discrepancy-oriented information through the adaptive combination of *C* and *M*. This residual organization prevents the final feature from depending entirely on the estimated selection map and allows CCE to refine an existing fused representation rather than reconstructing it from the two descriptors alone. The resulting $F_{\mathrm{out}}\in R^{C\times H\times W}$ is subsequently passed to the multi-scale detection head.

## 3. Results

### 3.1. Datasets

Experiments were conducted on two paired VIS–SAR remote sensing datasets, OGSOD-1.0 [44] and SpaceNet6-OTD-Fog. The two datasets provide complementary evaluation settings in terms of object categories, target distributions, and visible-image quality. OGSOD-1.0 contains multiple categories acquired over diverse scenes, whereas SpaceNet6-OTD-Fog focuses on oil-tank detection under varying target densities and degraded visible observations.

The training and testing partitions adopted in this study follow the predefined split protocols reported in the corresponding dataset studies. No additional random splitting or reassignment of image patches was performed. For each paired VIS–SAR sample, both modalities were consistently retained within the same partition. The dataset statistics reported in Table 3 correspond to the paired samples used in our experiments.

**Figure 5:**
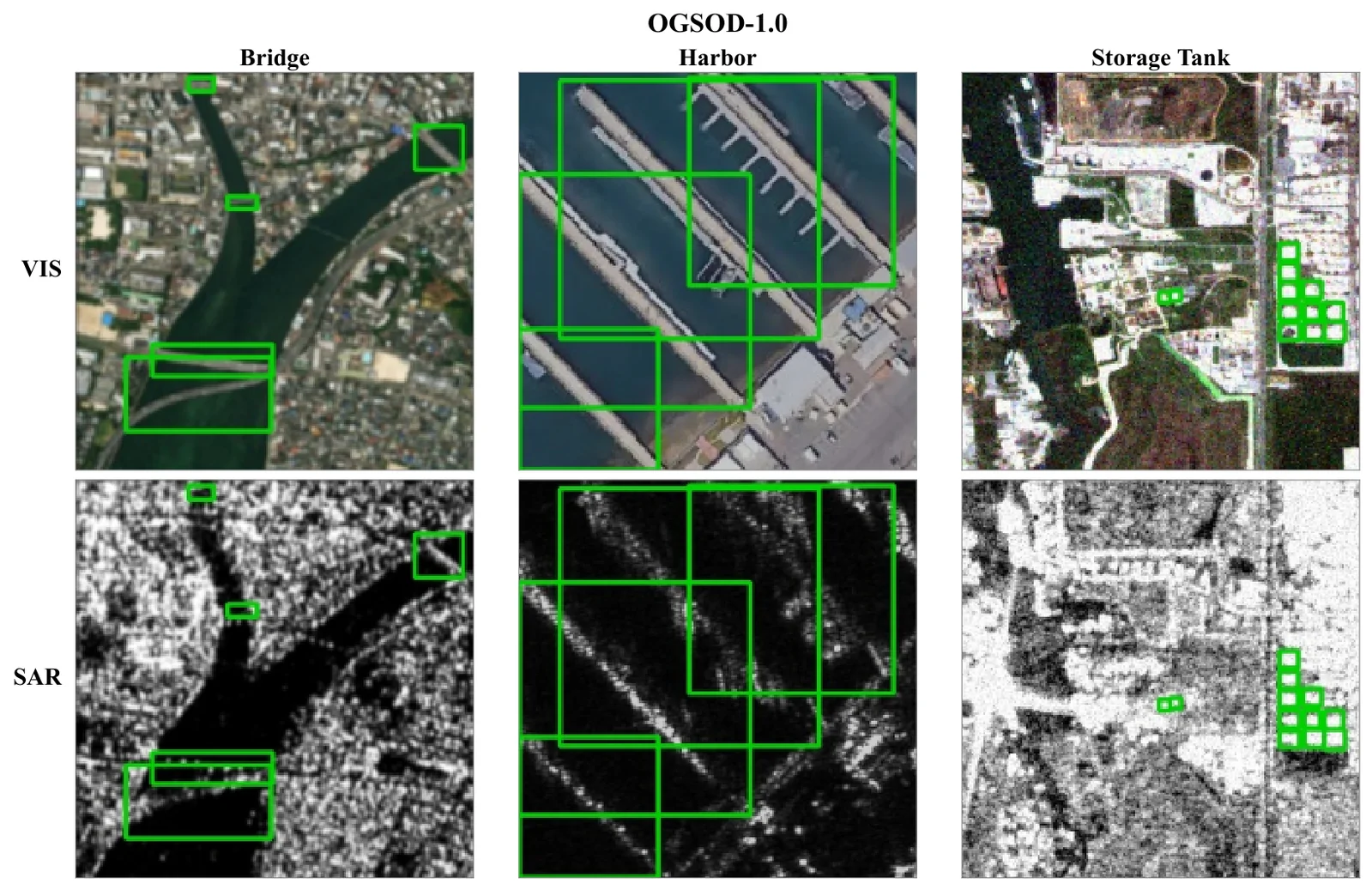
Representative paired VIS and SAR samples from OGSOD-1.0. The columns show Bridge, Harbor, and Storage Tank scenes, respectively. Green boxes denote ground-truth annotations.

OGSOD-1.0 is a paired optical–SAR benchmark constructed for remote sensing object detection. Its SAR imagery was acquired by the Gaofen-3 satellite, while the corresponding visible imagery was collected from Google Earth. The two modalities were spatially aligned and provided as 256×256 image patches. Three object categories are annotated: Bridge, Harbor, and Storage Tank. As illustrated in Figure 5, the paired observations exhibit substantial differences in appearance. Visible images preserve rich texture and structural details, whereas SAR images primarily reflect target scattering responses and contain more pronounced speckle-like background fluctuations. These cross-modal differences make OGSOD-1.0 suitable for evaluating feature interaction across heterogeneous remote sensing observations.

**Figure 6:**
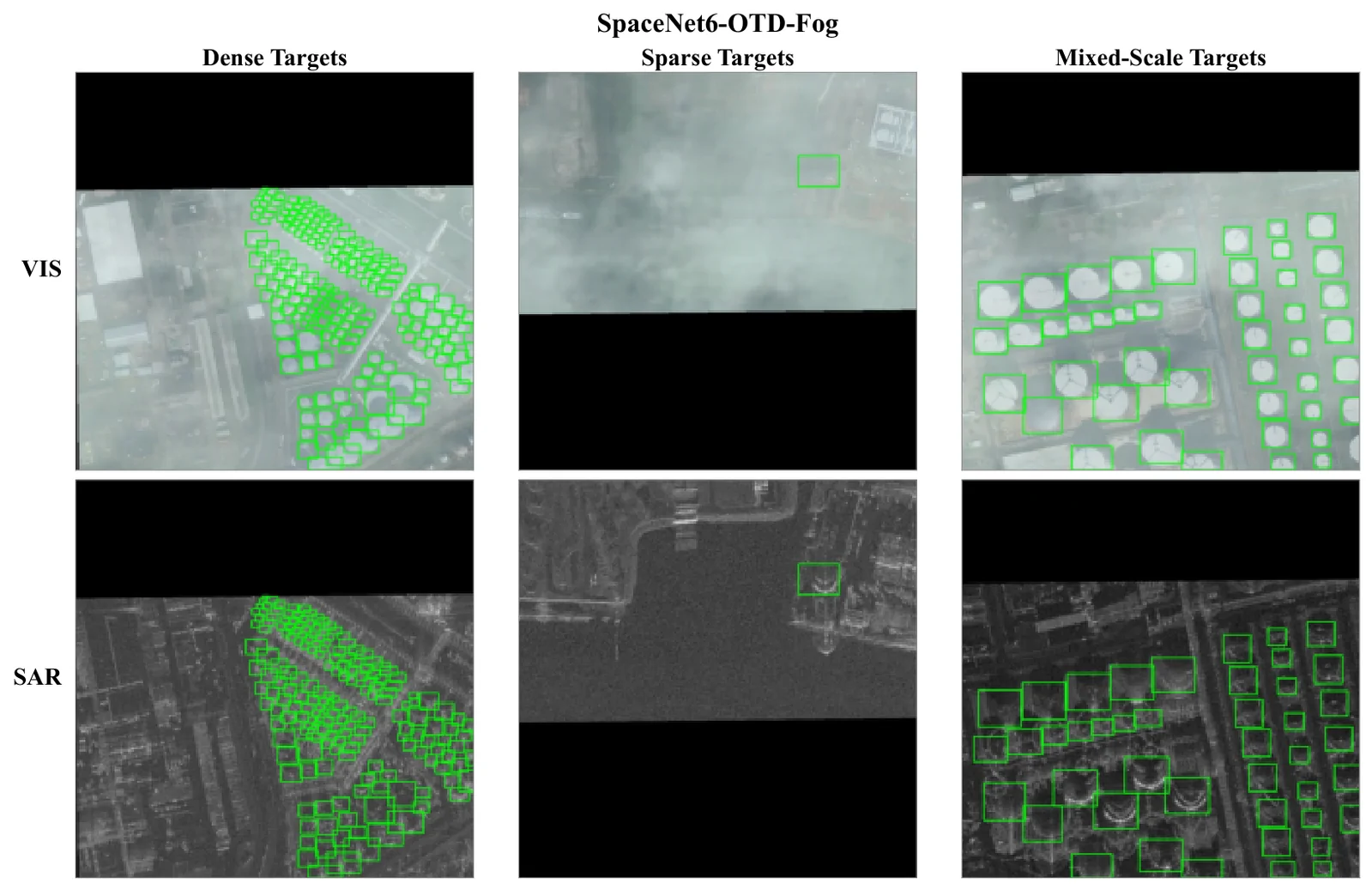
Representative paired VIS and SAR samples from SpaceNet6-OTD-Fog. The columns show dense-target, sparse-target, and mixed-scale oil-tank scenes, respectively. Green boxes denote ground-truth annotations.

SpaceNet6-OTD-Fog is an oil-tank detection dataset derived from SpaceNet6-OTD [45], which was constructed from the multi-sensor SpaceNet6 dataset [46]. The version used in this study contains 622 training pairs and 200 testing pairs, with Oil Tank as the single detection category. In contrast to the category diversity of OGSOD-1.0, this dataset presents substantial variations in target density and scale within the same object class. Moreover, the visible observations are affected by fog, leading to reduced contrast and partial loss of appearance information. Representative examples are shown in Figure 6, including densely distributed, sparsely distributed, and mixed-scale oil-tank scenes. Dense target arrangements also produce visually complex responses in SAR imagery, providing an additional challenge for reliable cross-modal interaction.

Together, the two datasets evaluate the proposed method from different perspectives. OGSOD-1.0 emphasizes cross-category VIS–SAR representation under diverse remote sensing scenes, while SpaceNet6-OTD-Fog provides a more focused setting for examining cross-modal fusion under changes in target density, scale, and visible-image degradation.

### 3.2. Experimental Settings

All experiments were implemented in PyTorch and conducted on a workstation equipped with four NVIDIA GeForce RTX 3090 GPUs, each with 24 GB of memory, and an Intel Xeon Gold 6530 CPU. The software environment consisted of Python 3.10.20, PyTorch 2.5.1, and CUDA 12.1.

The input resolution was fixed at 640×640. All models were trained from scratch for 400 epochs with a batch size of 64. Stochastic gradient descent (SGD) was adopted as the optimizer, with an initial learning rate of 0.01 and a weight decay of $5\times 10^{-4}$. A cosine learning-rate schedule was used throughout training. The default data augmentation settings of the adopted YOLO framework were retained without additional dataset-specific augmentation. The main training settings are summarized in Table 4.

The same training configuration was applied to OGSOD-1.0 and SpaceNet6-OTD-Fog. All ablation variants were also trained under identical settings, with the random seed fixed to 42. This protocol reduces variations introduced by differences in optimization and training configurations, allowing the effects of the investigated architectural components to be evaluated under controlled conditions.

Regarding model selection, all models were trained for a fixed 400 epochs, and the checkpoint saved at the final epoch (last.pt) was used for the reported results. Therefore, checkpoint selection was determined by the predefined training duration rather than by the highest evaluation score. No separate validation subset was created. In the dataset configuration, the predefined testing partition was also assigned to the validation entry for periodic metric computation during training. However, these evaluation metrics were not used to select checkpoints or determine the reported results. The same fixed-epoch checkpoint selection protocol was applied consistently to all baseline models and ablation experiments.

### 3.3. Evaluation Metrics

Detection performance was evaluated using Precision (*P*), Recall (*R*), average precision (AP), and mean average precision (mAP). The intersection over union (IoU) between a predicted bounding box $B_{p}$ and its corresponding ground-truth box $B_{g}$ is defined as

(16) $$\mathrm{IoU}=\frac{|B_{p}\cap B_{g}|}{|B_{p}\cup B_{g}|},$$

where |·| denotes the area of a bounding box or region. Precision and Recall are calculated as

(17) $$\begin{array}{cc} P & =\frac{TP}{TP+FP},\\ R & =\frac{TP}{TP+FN}, \end{array}$$

where TP, FP, and FN denote the numbers of true positives, false positives, and false negatives, respectively.

For each object category, AP is obtained from the area under the precision–recall curve. The mean AP over all $N_{c}$ categories is expressed as

(18) $$\mathrm{mAP}=\frac{1}{N_{c}}\sum_{i=1}^{N_{c}}{\mathrm{AP}}_{i},$$

where ${\mathrm{AP}}_{i}$ denotes the AP of the *i*-th category. In the following experiments, ${\mathrm{mAP}}_{50}$ denotes mAP evaluated at an IoU threshold of 0.50, while ${\mathrm{mAP}}_{50:95}$ represents the average mAP over IoU thresholds from 0.50 to 0.95 with an interval of 0.05. The latter provides a stricter assessment of localization quality across different matching criteria.

In addition to detection accuracy, the number of model parameters (Params) and floating-point operations (GFLOPs) were used to characterize model complexity. Params reflects the number of trainable parameters in the model, whereas GFLOPs measures the computational cost of a forward pass under a given input resolution. For a convolutional layer, the number of floating-point operations can be expressed as

(19) $${\mathrm{FLOPs}}_{\mathrm{conv}}=2H_{o}W_{o}C_{o}\left(\frac{C_{i}}{g}K_{h}K_{w}\right),$$

where $H_{o}$ and $W_{o}$ denote the spatial dimensions of the output feature map, $C_{i}$ and $C_{o}$ are the numbers of input and output channels, $K_{h}$ and $K_{w}$ represent the convolution kernel size, and *g* denotes the number of groups. The factor of 2 accounts for one multiplication and one addition. Accordingly, the overall computational complexity of a network is reported as

(20) $$\mathrm{GFLOPs}=10^{-9}\sum_{l=1}^{L}{\mathrm{FLOPs}}_{l},$$

where ${\mathrm{FLOPs}}_{l}$ denotes the floating-point operations of the *l*-th computational layer. Params and GFLOPs are considered together with detection accuracy in the subsequent comparisons.

### 3.4. Experimental Results

#### 3.4.1. Comparison with State-of-the-Art Methods

To evaluate the detection performance of the proposed method, comparisons were organized into three groups: single-modal detectors, basic VIS–SAR fusion baselines, and representative multimodal detection methods. On OGSOD-1.0, YOLOv5n, YOLOv8n, YOLO11n, and RT-DETR-L were evaluated separately using VIS and SAR inputs. RT-DETR-L, with 32.0 M parameters and 103.4 GFLOPs, is included as a high-capacity single-modal reference for each modality. We did not construct a dual-branch RT-DETR-L variant; therefore, its results are provided for modality-specific reference rather than as an architecture- or capacity-matched comparison with the proposed lightweight detector.

For a controlled evaluation of multimodal fusion, YOLO11-RGBT and YOLO11-RGBT-Add were included as basic dual-branch baselines. Both use the same YOLO11n backbone, multi-scale neck, and detection head as the proposed model, with differences confined to their feature-fusion operations. SuperYOLO, ICAFusion, and YOLO-CMFM were further included as representative VIS–SAR detection methods. The quantitative results are summarized in Table 5.

The single-modal results first reveal a consistent performance gap between VIS and SAR observations on OGSOD-1.0. For the four evaluated detectors, the VIS-based variants achieve mAP_50_ values of 83.7%, 84.8%, 86.2%, and 88.2%, respectively, all higher than their SAR-based counterparts. A similar trend is observed for mAP_50:95_. For example, YOLO11n obtains 86.2% mAP_50_ and 48.2% mAP_50:95_ using VIS images, compared with 85.3% and 46.4% using SAR images. These results indicate that the two modalities provide substantially different levels of discriminative information when used independently. Nevertheless, the SAR-only models remain competitive on several categories, suggesting that SAR observations contain target cues that are complementary rather than redundant to the VIS modality.

Introducing both modalities leads to a clear improvement over single-modal detection. The YOLO11-RGBT baseline reaches 90.3% mAP_50_ and 52.1% mAP_50:95_, improving over the VIS-only YOLO11n by 4.1 and 3.9 percentage points, respectively. In contrast, direct element-wise addition in YOLO11-RGBT-Add results in 88.5% mAP_50_ and 51.3% mAP_50:95_, which are lower than those of the original RGBT baseline. This observation suggests that simply combining features from the two modalities does not necessarily lead to more effective multimodal representations. The interaction mechanism therefore plays an important role in determining how complementary information is preserved and integrated.

The dedicated multimodal methods further improve upon the basic fusion baselines. SuperYOLO, ICAFusion, and YOLO-CMFM obtain mAP_50_ values of 93.7%, 93.4%, and 93.9%, respectively. Among the compared methods, YOLO-CMFM provides the strongest overall competing result, reaching 55.9% mAP_50:95_. The proposed method increases these two metrics to 95.1% and 56.8%, corresponding to improvements of 1.2 and 0.9 percentage points over YOLO-CMFM. Improvements are also observed across all three object categories. The proposed model achieves AP_50_ values of 95.7%, 98.7%, and 90.9% for Bridge, Harbor, and Storage Tank, respectively. In particular, the Storage Tank result exceeds the second-best value of 90.4% obtained by SuperYOLO, indicating that the overall improvement is not restricted to a single object category.

The proposed model also retains a relatively compact architecture. It contains 4.6 M parameters and requires 10.4 GFLOPs at an input resolution of 640×640. These values remain close to the 3.7 M parameters and 9.3 GFLOPs of the YOLO11-RGBT baseline, while being substantially lower than those of ICAFusion and YOLO-CMFM.

To further examine whether the observed improvements generalize to a different VIS–SAR setting, experiments were also conducted on SpaceNet6-OTD-Fog. Considering that this dataset contains a single object category, YOLO11n was used as the representative single-modal detector for both VIS and SAR inputs, while the same multimodal baselines and fusion methods were retained. The results are summarized in Table 6.

A larger difference between the two single modalities can be observed on SpaceNet6-OTD-Fog. VIS-based YOLO11n achieves 62.8% mAP_50_ and 34.6% mAP_50:95_, whereas the SAR-only counterpart obtains 56.2% and 32.3%, respectively. The SAR-only model also exhibits a considerably lower Recall of 42.0%, compared with 55.1% for the VIS-based model. This indicates that relying on SAR observations alone leads to more missed targets under this dataset setting. After incorporating both modalities, the YOLO11-RGBT baseline increases mAP_50_ to 67.9% and mAP_50:95_ to 36.5%, confirming that joint VIS–SAR representation remains beneficial on this dataset.

The comparison among multimodal approaches shows a trend consistent with that observed on OGSOD-1.0. SuperYOLO, ICAFusion, and YOLO-CMFM achieve mAP_50_ values between 68.8% and 69.9%, whereas the proposed method reaches 70.6%. For the stricter mAP_50:95_ metric, the proposed model achieves 38.5%, outperforming YOLO-CMFM by 0.9 percentage points. It also obtains the highest Recall of 64.2%. Although its Precision of 85.3% is slightly lower than the 85.6% of YOLO-CMFM, the higher Recall and AP metrics indicate a better overall balance between target retrieval and localization quality.

Across the two datasets, the results show that multimodal detection consistently benefits from combining VIS and SAR observations, but the magnitude of the gain depends strongly on the fusion strategy. Basic feature addition provides only limited improvement, whereas dedicated cross-modal interaction methods achieve substantially stronger results. The proposed model attains the highest mAP_50_ and mAP_50:95_ on both datasets while maintaining a relatively small computational overhead, supporting the effectiveness of the proposed cross-modal interaction design under different object categories, target distributions, and VIS observation conditions.

#### 3.4.2. Ablation Study

The comparative results in Section 3.4.1 demonstrate the overall effectiveness of the proposed model. To further identify the source of the performance gains and examine the contributions of the individual components, a complete ablation study was conducted on OGSOD-1.0. All eight combinations of MCD, DLBCA, and CCE were evaluated under the same training configuration described in Section 3.2. The YOLO11-RGBT model without the three proposed components was used as the baseline. The results are reported in Table 7.

When evaluated individually, all three components improve the two mAP metrics over the baseline. MCD increases mAP50 from 90.3% to 91.8% and mAP50:95 from 52.1% to 52.8%. DLBCA further raises these metrics to 92.2% and 53.6%, corresponding to gains of 1.9 and 1.5 percentage points over the baseline. Although DLBCA introduces additional learnable parameters, its low-bandwidth design replaces part of the original fusion computation with spatially reduced key–value interaction.As a result, the DLBCA-only variant slightly reduces the overall computational cost, from 9.3 to 9.2 GFLOPs. CCE achieves the highest mAP50 among the single-component variants, reaching 92.5%, while its mAP50:95 reaches 53.3%. The consistent improvements obtained by the three individual components indicate that each stage contributes useful information to the overall fusion process. In particular, the improvement introduced by MCD is consistent with the benefit of refining the SAR representation before cross-modal interaction, whereas the gains from DLBCA and CCE support the usefulness of selective cross-modal exchange and subsequent feature enhancement, respectively.

The pairwise configurations provide further evidence of complementarity among the proposed components. Combining MCD with DLBCA increases mAP_50_ and mAP_50:95_ to 93.7% and 54.1%, respectively. The MCD+CCE configuration obtains 92.6% mAP_50_ and 55.5% mAP_50:95_. Compared with CCE alone, its improvement at IoU = 0.5 is limited, whereas the gain under the stricter mAP_50:95_ criterion reaches 2.2 percentage points. This difference suggests that the benefit of combining the two components becomes more apparent when detection performance is evaluated across multiple IoU thresholds. Among the pairwise variants, DLBCA+CCE performs best, achieving 94.7% mAP_50_ and 56.1% mAP_50:95_, which are 4.4 and 4.0 percentage points higher than the baseline. This result indicates a particularly strong complementarity between cross-modal interaction and the subsequent consensus–complementary enhancement stage.

The complete configuration integrating MCD, DLBCA, and CCE achieves the highest overall detection performance, with 95.1% mAP_50_ and 56.8% mAP_50:95_. Relative to the baseline, the improvements reach 4.8 and 4.7 percentage points, respectively. The full model also improves upon the strongest pairwise configuration, DLBCA+CCE, by 0.4 percentage points in mAP_50_ and 0.7 percentage points in mAP_50:95_. Although the MCD+DLBCA variant yields a slightly higher Precision of 90.0%, the complete model achieves the highest Recall of 88.3% together with the best AP-based metrics. Therefore, its advantage is reflected in the overall detection performance rather than in a single operating-point metric.

The accuracy gains are obtained with a moderate increase in model complexity. Compared with the baseline, the complete model increases the number of parameters from 3.7 M to 4.6 M and the computational cost from 9.3 to 10.4 GFLOPs. Thus, the addition of the three components introduces approximately 0.9 M parameters and 1.1 GFLOPs while providing gains of 4.8 and 4.7 percentage points in mAP_50_ and mAP_50:95_, respectively. A more detailed analysis of the accuracy–complexity trade-off is presented in Section 3.4.7.

Overall, the ablation results are consistent with the staged design of LB-CIM. MCD improves the SAR representation before multimodal interaction, DLBCA performs selective low-bandwidth information exchange between the two modalities, and CCE further reorganizes the interacted features according to their consensus and complementary content. The progressive improvement from individual components to pairwise combinations and finally to the complete configuration indicates that the three stages provide complementary rather than redundant contributions to VIS–SAR feature fusion.

#### 3.4.3. Sensitivity Analysis of Internal LB-CIM Configurations

**MCD Pooling-Scale Sensitivity.**

To investigate the effect of pooling-scale selection on the performance of the Multi-Scale Consistency Denoising (MCD) module, we conduct a sensitivity analysis using different average-pooling kernel configurations.

Specifically, three configurations are evaluated:

1. A single-scale configuration with a 3×3 average-pooling kernel.
2. The proposed dual-scale configuration with 3×3 and 5×5 average-pooling kernels.
3. A larger-scale configuration with 5×5 and 7×7 average-pooling kernels.

For the single-scale variant, the cross-scale discrepancy input is removed, while the gating network architecture is retained to ensure a consistent parameterization. All other network components and training settings remain unchanged. The results are reported in Table 8.

As shown in Table 8, the proposed 3×3+5×5 configuration achieves the best performance across all four evaluation metrics. Compared with the single-scale 3×3 variant, the proposed configuration improves precision, recall, mAP_50_, and mAP_50:95_ by 0.6, 0.8, 0.6, and 0.7 percentage points, respectively. This improvement indicates that incorporating cross-scale discrepancy information provides additional cues for adaptive noise suppression beyond those captured by local residuals alone.

We further evaluate a larger pooling configuration using 5×5 and 7×7 kernels. Although this configuration retains the multi-scale structure, its performance decreases compared with the proposed setting, with mAP_50_ and mAP_50:95_ dropping by 0.8 and 1.1 percentage points, respectively. This may be attributed to the excessive smoothing introduced by larger pooling windows, which can weaken local structural variations and fine-grained responses associated with small targets in SAR imagery. Consequently, the discrepancy cues may become less effective in distinguishing informative local responses from noise-related variations.

All three configurations maintain the same reported parameter count of 4.6 M and computational cost of 10.4 GFLOPs. Therefore, the observed performance differences are primarily associated with the choice of pooling scales rather than changes in model capacity or reported computational complexity.

Overall, these results support the use of the 3×3+5×5 configuration in MCD, as it provides a more favorable balance between local detail preservation and multi-scale contextual characterization among the evaluated settings.

**DLBCA Token Reduction Sensitivity.**

To investigate the effect of spatial token compression on cross-modal interaction, we evaluate three reduction ratios (R=1, 2, and 4) in DLBCA, while keeping the remaining architectural components and training settings unchanged. The reduction ratio controls the spatial compression applied to the key and value representations, whereas the query representations retain their original spatial resolution. Specifically, R=1 preserves all spatial tokens without compression, R=2 reduces the spatial dimensions by a factor of two, and R=4 applies more aggressive compression. The same reduction ratio is consistently applied at P3, P4, and P5. The experimental results are summarized in Table 9.

As shown in Table 9, the reduction ratio introduces a clear trade-off between detection accuracy and computational cost. Without spatial compression (R=1), DLBCA achieves the highest detection performance, with 95.3% mAP_50_ and 57.2% mAP_50:95_. However, this configuration requires 12.9 GFLOPs due to the retention of the complete spatial token representations.

When the reduction ratio is increased to R=2, the mAP_50_ and mAP_50:95_ decrease slightly by only 0.2 and 0.4 percentage points, respectively, while the computational cost is reduced from 12.9 to 10.4 GFLOPs, corresponding to a reduction of approximately 19.4%. This indicates that moderate spatial compression can substantially reduce the computational burden while preserving most of the detection performance.

With more aggressive compression (R=4), the computational cost is further reduced to 9.9 GFLOPs. However, this reduction provides only a limited computational saving of approximately 4.8% compared with R=2, while mAP_50_ and mAP_50:95_ decrease by 0.8 and 1.1 percentage points, respectively. The decline in detection performance suggests that excessive spatial compression may discard useful spatial correspondence information, thereby weakening cross-modal feature interaction, particularly when fine-grained spatial details are important for object localization.

Overall, the results demonstrate that R=2 provides a favorable balance between detection accuracy and computational efficiency. Compared with the uncompressed configuration, it achieves a substantial reduction in computational cost with only minor performance degradation. Meanwhile, further increasing the compression ratio to R=4 results in a more noticeable accuracy loss with limited additional computational savings. Therefore, R=2 is adopted as the default reduction ratio in the proposed DLBCA module.

**CCE Descriptor Construction Analysis.**

To evaluate the effectiveness of the descriptor construction strategy in CCE, we compare element-wise multiplication (Ours) with cosine similarity. In both configurations, modality-specific LayerNorm, complementary descriptor construction, adaptive selection, and subsequent refinement remain unchanged. Only the product-based descriptor construction is replaced to ensure a controlled comparison.

For the proposed method, the product-based descriptor is constructed through element-wise multiplication:

(21) $$P_{\mathrm{prod}}={\bar{Z}}_{v}⊙{\bar{Z}}_{s},$$

where ${\bar{Z}}_{v}$ and ${\bar{Z}}_{s}$ denote the normalized visible and SAR features, respectively. For comparison, cosine similarity is calculated along the channel dimension at each spatial location and mapped to [0,1]:

(22) $$w_{\cos}=\frac{1}{2}\left(1+\frac{\langle {\bar{Z}}_{v},{\bar{Z}}_{s}\rangle }{∥{\bar{Z}}_{v}{∥}_{2}{∥{\bar{Z}}_{s}∥}_{2}+\epsilon }\right),$$

(23) $$P_{\cos}=w_{\cos}⊙\frac{{\bar{Z}}_{v}+{\bar{Z}}_{s}}{2},$$

where $w_{\cos}$ is a spatial similarity map shared across channels. Both descriptors are subsequently processed by the same consensus refinement branch. The complementary descriptor construction, adaptive selection, and other network components remain unchanged. The results are reported in Table 10.

As shown in Table 10, element-wise multiplication outperforms cosine similarity across all four detection metrics, improving precision, recall, mAP_50_, and mAP_50:95_ by 0.4, 0.7, 0.6, and 0.7 percentage points, respectively, while maintaining the same reported computational cost of 10.4 GFLOPs.

This difference is related to the response representations produced by the two operations. Since LayerNorm outputs contain both positive and negative responses, the element-wise product encodes their polarity relationships. Specifically, positive products indicate same-polarity responses, including both positive-positive and negative-negative patterns, whereas negative products indicate opposite-polarity responses. Thus, the product-based descriptor preserves signed, channel-wise cross-modal relationships rather than directly indicating target presence or absence. The subsequent depthwise convolution and nonlinear activation further refine these interaction patterns.

In contrast, cosine similarity produces a single spatial weight shared across all channels. Although the averaged feature retains its channel dimension, the shared weighting cannot independently characterize channel-specific relationships, potentially limiting the representation of fine-grained cross-modal details.

Overall, element-wise multiplication provides a more effective polarity-aware descriptor for the current CCE design by preserving channel-wise response relationships and achieving better detection performance without increasing the reported computational cost. Therefore, it is retained as the default descriptor construction strategy.

#### 3.4.4. Mechanism Analysis of DLBCA

To further isolate the contributions of the internal components of DLBCA, we conduct mechanism-level ablation experiments on OGSOD-1.0. As shown in Table 11, three factors are examined: the transferred value representation, the difference-aware gate, and the direction of cross-modal interaction. MCD and CCE are retained in all variants, while the remaining training settings are kept unchanged. For a consistent model configuration, the gate module and its parameters are retained in all variants. In variants marked as “No”, the computed gate outputs are bypassed and do not modulate the differential updates, whereas in variants marked as “Yes”, the gate outputs are applied to the corresponding updates. Consequently, all variants maintain the same parameter count of 4.6 M and nominal computational complexity of 10.4 GFLOPs. These comparisons are designed to examine the selected mechanisms under specific interaction settings.

Rather than exhaustively evaluating all possible combinations, the variants are organized into controlled comparisons to examine the three design choices under their relevant settings: A versus B for value representation, B versus E for gate application, and C, D, and E for interaction direction.

First, we examine the contribution of differential-value transfer independently of the gating operation. Variants A and B both employ bidirectional interaction, while the gate outputs are bypassed in both cases. The only difference is the transferred value representation: Variant A uses the standard counterpart values, whereas Variant B adopts the proposed directional differential values. This modification improves mAP50 from 93.5% to 94.0% and mAP50:95 from 55.8% to 56.1%. The improvement indicates that differential-value transfer provides additional benefits even without applying the difference-aware gate.

We next examine the effect of applying the difference-aware gate. Variants B and E retain the same differential-value transfer and bidirectional interaction. Although the gate module is retained in both variants, its output is bypassed in Variant B and applied to the differential updates in Variant E. Enabling the gate increases mAP50 from 94.0% to 95.1% and mAP50:95 from 56.1% to 56.8%. Since the instantiated parameter count and nominal computational complexity remain unchanged, this improvement reflects the effect of applying the learned gating operation rather than an increase in the reported model size. The result suggests that regulating differential responses according to feature content is beneficial for cross-modal interaction. In particular, the gate allows the network to emphasize informative discrepancies while suppressing less reliable responses, which may arise from noise or residual registration errors. The gate does not explicitly correct geometric misregistration; instead, it adaptively regulates the resulting differential updates.

Since all three variants employ the difference-aware gate, this comparison focuses on the effect of interaction direction under the gated differential-value transfer setting.

Overall, the mechanism-level ablation provides complementary evidence for the three design choices in DLBCA. The comparison between Variants A and B demonstrates the benefit of differential-value transfer without applying the gate, while the comparison between Variants B and E further shows the benefit of enabling the difference-aware gating operation under bidirectional interaction. The comparison among Variants C, D, and E highlights the advantage of retaining both interaction directions when the gate is enabled. These results support the complementary roles of differential-value transfer, adaptive gating, and bidirectional information exchange in the proposed DLBCA.

#### 3.4.5. Ordering and Multi-Level Placement Analysis

We first examine the placement of MCD relative to cross-modal interaction. Without MCD, the DLBCA → CCE configuration achieves 94.7% mAP_50_ and 56.1% mAP_50:95_. When MCD is moved after DLBCA, the performance decreases slightly to 94.5% and 55.8%, respectively. In contrast, placing MCD before DLBCA yields the best performance of 95.1% mAP_50_ and 56.8% mAP_50:95_.

This result suggests that the effectiveness of MCD depends on its position in the interaction pipeline rather than merely on the presence of an additional refinement module. Before cross-modal interaction, MCD operates on the modality-specific SAR representation and can suppress potentially unreliable responses before they are propagated to the visible branch. When applied after DLBCA, however, the SAR representation already contains information transferred from the visible branch. SAR-specific residual suppression at this stage may attenuate useful interacted responses and disturb the paired representations subsequently processed by CCE. Therefore, the MCD → DLBCA → CCE ordering is adopted in the complete model.

We further investigate the contribution of applying LB-CIM at different pyramid levels. The P3, P4, and P5 features differ in spatial resolution and semantic abstraction, and therefore provide different contexts for cross-modal interaction. To isolate the effect of fusion placement, LB-CIM is selectively enabled at individual or combined pyramid levels. At levels where LB-CIM is not enabled, the baseline concatenation-based fusion followed by a 1×1 projection is retained. The backbone, detection neck, detection head, and all training settings are kept unchanged.

The single-level results show that all three pyramid levels can benefit from the proposed interaction module, but their contributions are different. Applying LB-CIM only at P3 achieves 92.8% mAP_50_ and 54.0% mAP_50:95_, outperforming the P4-only configuration (92.1%/53.5%) and the P5-only configuration (91.4%/52.9%). The stronger performance of P3 may be associated with its higher spatial resolution and richer local detail, which provide a more informative spatial basis for cross-modal interaction. In contrast, P5 contains more abstract semantic information but has substantially lower spatial resolution, which may partly explain the smaller gain observed when LB-CIM is applied at this level alone.

Combining adjacent pyramid levels further improves the detection performance. The P3 + P4 configuration reaches 94.4% mAP_50_ and 55.9% mAP_50:95_, corresponding to gains of 1.6 and 1.9 percentage points, respectively, over the P3-only configuration. Similarly, adding P5 to P4 improves the performance from 92.1%/53.5% to 92.9%/54.2%. These results indicate that cross-modal interactions at different pyramid levels provide complementary spatial and semantic information rather than fully redundant representations.

The complete P3 + P4 + P5 configuration achieves the best performance, with 95.1% mAP_50_ and 56.8% mAP_50:95_. Compared with the P3 + P4 configuration, introducing LB-CIM at P5 provides an additional improvement of 0.7 percentage points in mAP_50_ and 0.9 percentage points in mAP_50:95_. Although P5 provides the smallest improvement when used alone, its inclusion in the complete multi-level configuration still contributes additional semantic information. These observations support the use of LB-CIM at all three pyramid levels in the final model, while also showing that the contribution of each level is not uniform.

Taken together, the ordering experiment in Table 12 and the pyramid-level placement experiment in Table 13 show that both the internal processing order and the pyramid-level placement affect the effectiveness of LB-CIM. The MCD → DLBCA → CCE ordering and the joint P3 + P4 + P5 placement provide the most effective configuration among the settings evaluated in this work.

#### 3.4.6. Generalization Across Different YOLO Architectures

The ablation results in Section 3.4.2 verify that MCD, DLBCA, and CCE provide complementary contributions within the YOLO11-RGBT baseline. We further investigate whether the effectiveness of LB-CIM is tied to this specific architecture or can be transferred to other YOLO-based multimodal detectors. To this end, LB-CIM was integrated into YOLOv5n-RGBT, YOLOv8n-RGBT, and YOLO11n-RGBT under the same experimental protocol on OGSOD-1.0. Each architecture was evaluated before and after introducing LB-CIM. The results are summarized in Table 14.

As shown in Table 14, introducing LB-CIM consistently improves detection accuracy across all three YOLO architectures. For YOLOv5n-RGBT, mAP_50_ increases from 86.1% to 89.3%, while mAP_50:95_ rises from 49.6% to 52.4%, corresponding to gains of 3.2 and 2.8 percentage points, respectively. A similar improvement is observed for YOLOv8n-RGBT, where the two metrics increase by 3.8 and 3.2 percentage points. These results indicate that the contribution of LB-CIM is not confined to the YOLO11n-based detector.

The largest improvement is observed when LB-CIM is integrated into YOLO11n-RGBT. Its mAP_50_ increases from 90.3% to 95.1%, and mAP_50:95_ improves from 52.1% to 56.8%, yielding gains of 4.8 and 4.7 percentage points, respectively. Although the magnitude of the gain varies among the three architectures, both AP metrics improve consistently in every case. This consistency suggests that the proposed interaction scheme can be adapted to different YOLO feature extraction and aggregation structures without relying on a single backbone configuration.

The improved accuracy is accompanied by a relatively stable increase in model complexity. Incorporating LB-CIM adds approximately 0.8–1.0 M parameters and 0.9–1.1 GFLOPs across the three architectures. Specifically, the parameter counts increase from 3.7 M to 4.7 M for YOLOv5n, from 4.4 M to 5.2 M for YOLOv8n, and from 3.7 M to 4.6 M for YOLO11n. The corresponding computational increments remain close to 1 GFLOP. The similar overhead across architectures indicates that the additional cost introduced by LB-CIM remains controlled when it is transferred to different YOLO variants.

Together with the module-level ablation results, these experiments provide evidence from two complementary perspectives. Section 3.4.2 shows that the three stages of LB-CIM contribute jointly to the final performance, whereas the present comparison demonstrates that the resulting fusion mechanism is not restricted to a single YOLO generation. These results support the architectural generalizability of LB-CIM and motivate the subsequent analysis of its accuracy–complexity trade-off.

#### 3.4.7. Complexity Analysis

**Figure 7:**
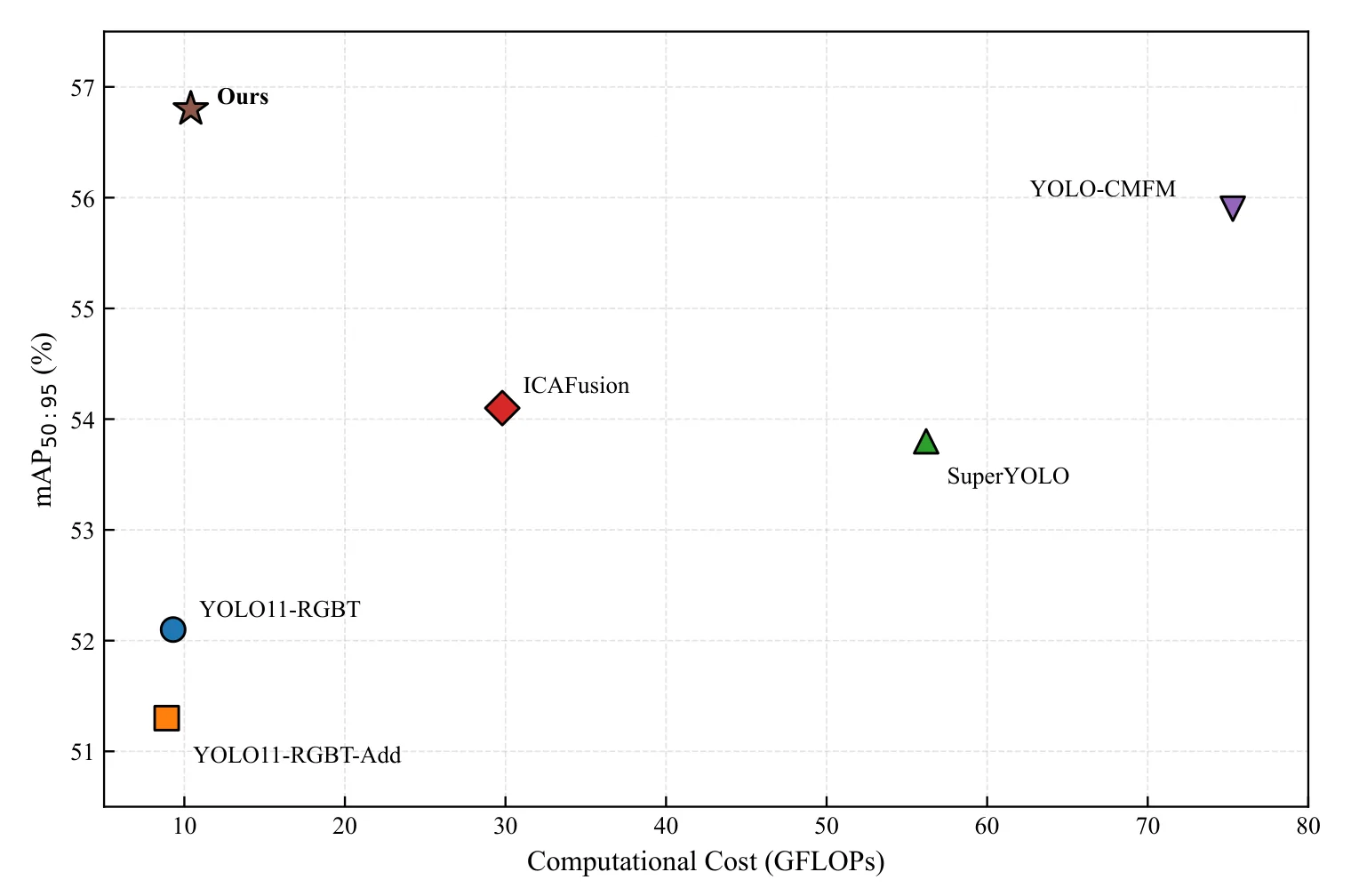
Accuracy–complexity comparison of different VIS–SAR detection methods on OGSOD-1.0.

Following the cross-architecture evaluation, we further examine the relationship between detection accuracy, model capacity, and computational cost through three complementary analyses: FLOPs-based accuracy comparison, capacity-matched conventional fusion comparison, and practical inference efficiency measurements. Figure 7 plots mAP_50:95_ against GFLOPs for the main VIS-SAR detection methods evaluated on OGSOD-1.0.

As shown in Figure 7, the proposed method achieves the highest mAP_50:95_ while maintaining a computational cost close to that of the YOLO11-RGBT baseline. Specifically, the baseline requires 9.3 GFLOPs and achieves an mAP_50:95_ of 52.1%, whereas LB-CIM requires 10.4 GFLOPs and achieves 56.8%. Thus, an additional 1.1 GFLOPs is associated with a gain of 4.7 percentage points in mAP_50:95_. The mAP_50_ also increases from 90.3% to 95.1%, indicating improved detection performance at both IoU evaluation settings.

The advantage becomes more evident when compared with computationally intensive multimodal fusion methods. SuperYOLO requires 56.2 GFLOPs and achieves an mAP_50:95_ of 53.8%, while ICAFusion achieves 54.1% with 29.8 GFLOPs. YOLO-CMFM achieves 55.9% but requires 75.3 GFLOPs. In comparison, LB-CIM achieves 56.8% with only 10.4 GFLOPs, exceeding the result of YOLO-CMFM by 0.9 percentage points while requiring substantially less computation. A similar trend is observed in model size: LB-CIM contains 4.6 M parameters, fewer than the 20.2 M of ICAFusion and the 18.5 M of YOLO-CMFM.

Notably, YOLO11-RGBT-Add has a lower computational cost of 8.9 GFLOPs, but its mAP_50:95_ decreases to 51.3%. This comparison indicates that minimizing computational cost alone does not necessarily lead to more effective multimodal feature fusion. Instead, LB-CIM incurs additional computational cost while providing stronger detection performance.

To further distinguish the contribution of the proposed fusion mechanisms from the effect of increased model capacity, we introduce a parameter-matched conventional convolutional baseline, denoted as YOLO11-RGBT-CM. This model retains the original dual-branch backbone, neck, and detection head, while employing additional standard convolutional layers at the P4 and P5 fusion stages to obtain a parameter count close to that of LB-CIM. The resulting baseline contains 4.63 M parameters, compared with 4.60 M for LB-CIM. No MCD, DLBCA, or CCE operations are incorporated into this baseline, allowing the performance contribution of the proposed fusion design to be examined against conventional convolutional capacity expansion. The comparison results are presented in Table 15.

As shown in Table 15, YOLO11-RGBT-CM achieves 92.8% mAP_50_ and 54.0% mAP_50:95_, improving upon the original YOLO11-RGBT baseline by 2.5 and 1.9 percentage points, respectively. This result confirms that increasing model capacity through conventional convolutional operations can improve detection performance.

More importantly, YOLO11-RGBT-CM and LB-CIM have nearly identical parameter counts (4.63 M vs. 4.60 M, a difference of approximately 0.7%). Despite this close parameter match, YOLO11-RGBT-CM achieves only 92.8% mAP_50_ and 54.0% mAP_50:95_, whereas LB-CIM reaches 95.1% and 56.8%, respectively. Thus, LB-CIM provides additional gains of 2.3 and 2.8 percentage points in mAP_50_ and mAP_50:95_, respectively. Furthermore, YOLO11-RGBT-CM requires 10.91 GFLOPs, compared with 10.40 GFLOPs for LB-CIM. These results indicate that the performance advantage of LB-CIM cannot be attributed solely to increased model capacity or computational expenditure. Instead, the proposed MCD, DLBCA, and CCE mechanisms provide additional detection benefits beyond conventional convolutional feature fusion under a comparable parameter budget.

To further assess practical inference efficiency, we benchmark YOLO11-RGBT, YOLO11-RGBT-Add, and LB-CIM under identical hardware and inference settings. The measured inference latency, throughput, and peak GPU memory consumption are reported in Table 16.

All three models are evaluated on a single NVIDIA RTX 3090 using four-channel inputs of size 640×640, a batch size of 1, and FP32 precision. After 50 warm-up iterations, three rounds of 300 forward passes are performed for measurement. Data loading, preprocessing, and non-maximum suppression (NMS) are excluded from the timing process.

As shown in Table 16, LB-CIM has a mean forward latency of 11.16 ms and a throughput of 89.42 images/s. Compared with YOLO11-RGBT, its latency increases from 8.75 to 11.16 ms, while its throughput decreases from 109.36 to 89.42 images/s. The peak allocated GPU memory increases from 0.087 to 0.108 GiB. Similar trends are observed relative to YOLO11-RGBT-Add, which achieves a latency of 8.70 ms and a throughput of 115.04 images/s.

These measurements show that the accuracy improvements of LB-CIM are accompanied by additional inference cost. The proposed model therefore provides a trade-off between detection performance and inference efficiency rather than a reduction in latency or memory consumption. Nevertheless, it maintains a throughput of approximately 89 images/s under the specified single-GPU, batch-one forward-inference setting.

Overall, the complexity analysis indicates that LB-CIM achieves stronger detection performance at the cost of additional computation and inference latency. The capacity-matched comparison further demonstrates that the accuracy gains cannot be explained solely by increased model capacity, while the measured inference results characterize the associated deployment cost. These results provide a comprehensive assessment of the proposed method in terms of detection accuracy, model complexity, and practical inference efficiency.

#### 3.4.8. Detection Error and Qualitative Analysis

To quantitatively analyze the detection errors of the baseline and the proposed LB-CIM model, we compare their true positives (TP), false positives (FP), and false negatives (FN) on the OGSOD-1.0 test set under identical inference settings. A prediction is considered a true positive when it matches an unmatched ground-truth box of the same class with an intersection over union (IoU) of at least 0.50. The confidence threshold and NMS IoU threshold are set to 0.25 and 0.70, respectively. The results are summarized in Table 17.

As shown in Table 17, LB-CIM increases the number of true positives from 8251 to 8859, while reducing false positives from 2154 to 2070 and false negatives from 1363 to 755. Precision increases from 79.30% to 81.06%, and recall improves from 85.82% to 92.15%. The F1-score also increases from 82.43% to 86.25%. These results indicate that LB-CIM improves the balance between detecting existing targets and avoiding incorrect detections, with a more pronounced improvement in reducing missed detections.

The gap between mAP_50_ and mAP_50:95_ reflects the stricter localization requirements imposed by higher IoU thresholds in the latter metric. As reported in Table 5, LB-CIM improves the baseline by 4.8 and 4.7 percentage points under these two metrics, respectively, indicating that its performance gains are maintained under stricter localization criteria.

To further examine whether the detection improvements vary with scene density, the OGSOD-1.0 test images are grouped according to the number of ground-truth objects per image. Images containing 1–2, 3–5, and at least 6 objects are categorized as sparse, medium-density, and dense scenes, respectively. The same trained models, confidence threshold, NMS IoU threshold, and ground-truth matching criterion are used for all groups. The results are summarized in Table 18.

As shown in Table 18, LB-CIM improves precision, recall, and F1-score across all three scene-density groups. The number of false negatives decreases from 294 to 143 in sparse scenes, from 448 to 189 in medium-density scenes, and from 621 to 423 in dense scenes. The corresponding recall improvements are 4.30, 8.68, and 6.35 percentage points, respectively. These results indicate that the proposed method improves target detection across different levels of scene density, with the largest recall gain observed in medium-density scenes.

Dense scenes remain more challenging for both models, as reflected by their lower recall and F1-scores. Moreover, the reduction in false positives in this group is limited. Therefore, the results support improved target recovery across different scene densities, but do not imply that all types of detection errors are equally reduced.

**Figure 8:**
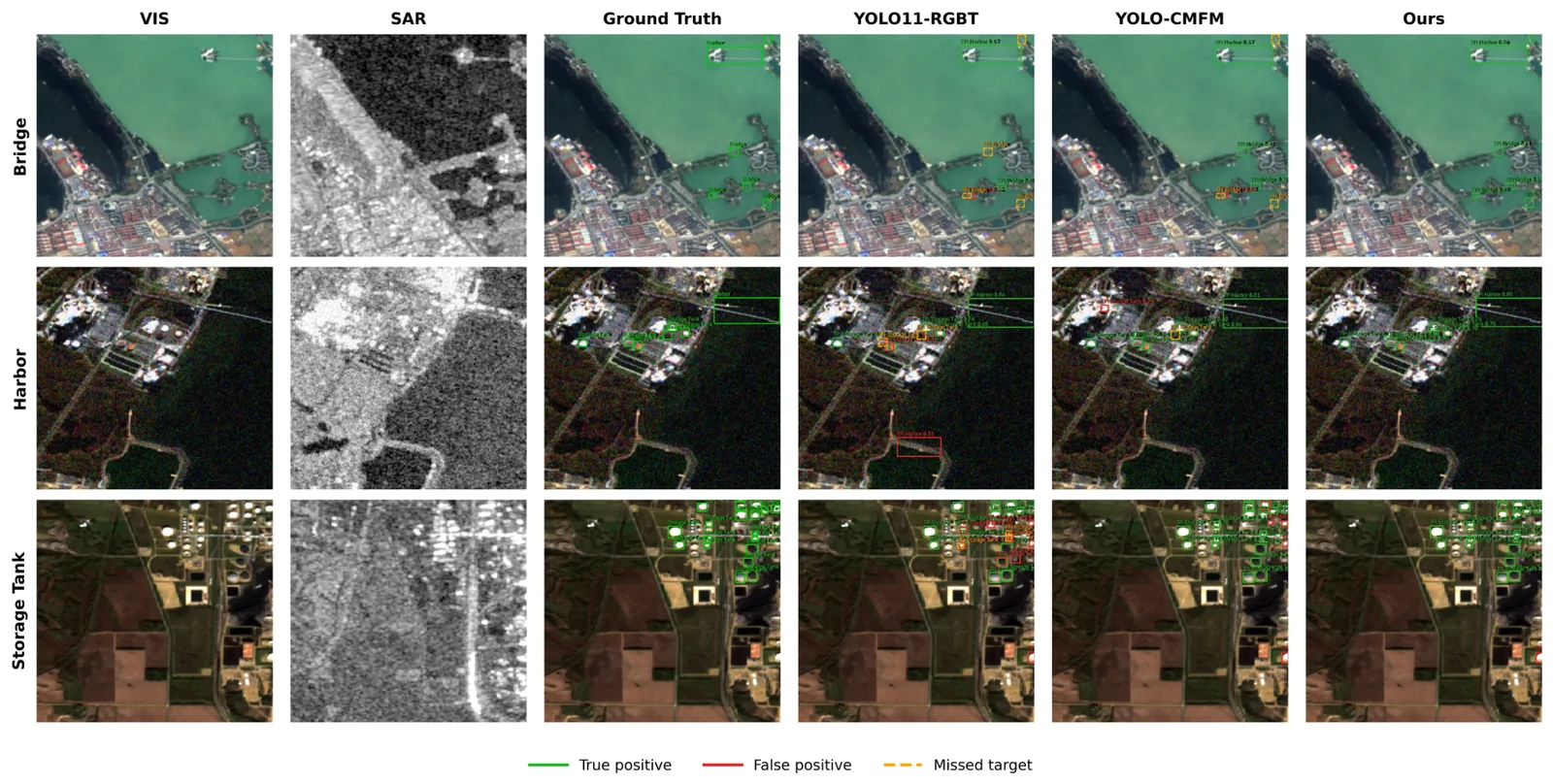
Qualitative detection comparison on the OGSOD-1.0 dataset. Representative bridge, harbor, and storage-tank scenes are shown from top to bottom. Green solid boxes indicate true positives, red solid boxes indicate false positives, and orange dashed boxes indicate missed targets.

**Figure 9:**
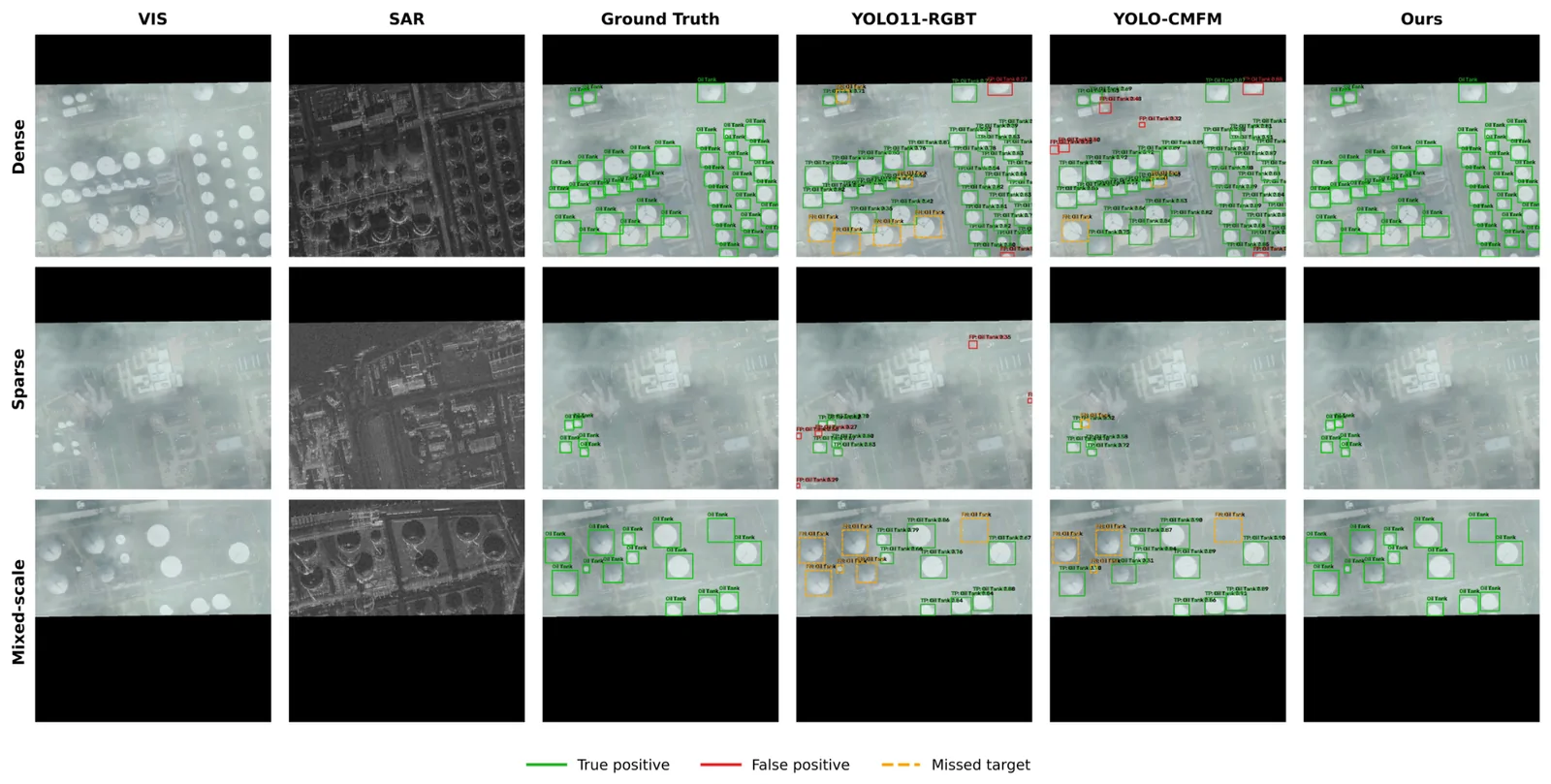
Qualitative detection comparison on the SpaceNet6-OTD-Fog dataset under dense, sparse, and mixed-scale target distributions. Green solid boxes indicate true positives, red solid boxes indicate false positives, and orange dashed boxes indicate missed targets.

To complement the aggregate error statistics with representative visual examples, Figure 8 and Figure 9 present qualitative comparisons on OGSOD-1.0 and SpaceNet6-OTD-Fog, respectively. The comparisons include the visible images, SAR images, ground-truth annotations, baseline predictions, YOLO-CMFM predictions, and the proposed method. Correct detections are indicated by green solid boxes, false positives by red solid boxes, and missed targets by orange dashed boxes.

Figure 8 shows representative examples from bridge, harbor, and storage-tank scenes in OGSOD-1.0. In the bridge scene, the YOLO11-RGBT baseline leaves several annotated targets undetected, while YOLO-CMFM recovers part of them but still exhibits missed detections. The proposed method provides a more complete set of detections in the same region. A similar difference can be observed in the harbor scene, where the baseline produces both missed detections and background false positives. YOLO-CMFM reduces part of these errors, whereas the proposed method yields a cleaner prediction with fewer unmatched responses. In the storage-tank scene, where multiple compact targets are distributed within a relatively dense area, the proposed method preserves more valid detections while reducing the missed and spurious responses observed in the comparison models.

The SpaceNet6-OTD-Fog examples in Figure 9 further illustrate the behavior of the different methods under dense, sparse, and mixed-scale target distributions. In the dense scene, both comparison models exhibit missed detections and false responses among closely distributed oil tanks, whereas the proposed method provides more complete target coverage. In the sparse scene, where only a limited number of valid targets are present against a low-contrast background, the proposed model produces a more concise set of predictions with fewer false alarms. For the mixed-scale example, the baseline and YOLO-CMFM still miss several targets of different spatial extents, while the proposed method maintains more consistent detections across the visible target scales.

These qualitative observations are consistent with the quantitative error analysis in Table 17 and Table 18. The overall statistics show reductions in missed detections and false positives, while the target-count-based analysis demonstrates improvements in precision, recall, and F1-score across all three groups. The examples in Figure 8 and Figure 9 provide complementary visual evidence of the detection behavior under different scene structures and target distributions.

## 4. Discussion

From a multimodal sensing perspective, the experimental results show that LB-CIM consistently improves detection performance across both VIS–SAR datasets while introducing only a moderate computational overhead. The results suggest that effective cross-modal fusion does not require unrestricted information exchange between heterogeneous sensing modalities; instead, selectively refining unreliable sensor responses and reducing redundant cross-modal interaction can provide a more efficient representation learning strategy.The consistent improvements across OGSOD-1.0 and SpaceNet6-OTD-Fog further suggest that the proposed fusion strategy remains effective under different object distributions and visible-image conditions.

The ablation results provide further insight into the contribution of the three stages in LB-CIM. MCD operates before cross-modal interaction and suppresses locally inconsistent SAR responses according to discrepancies observed across multiple spatial scales. This preprocessing does not assume that every high-frequency response corresponds to noise; instead, it attenuates regions that exhibit stronger local inconsistency while preserving the residual SAR structure. DLBCA then performs bidirectional cross-modal interaction using queries at the unreduced interaction-grid resolution and further spatially reduced key–value representations. Its differential value construction and difference-aware gating emphasize information that is not simply duplicated between the two modalities. Finally, CCE separates consensus and complementary cues after cross-modal interaction and adaptively combines them before generating the fused representation. The pairwise and full ablation results indicate that these stages are complementary rather than interchangeable, with the combination of DLBCA and CCE providing the strongest two-module configuration and the complete three-stage design yielding the best overall detection accuracy.

From a cross-modal representation perspective, the observed improvements are consistent with the staged interaction strategy adopted in LB-CIM. Direct feature addition provides only limited gains because modality-specific responses are mixed without explicitly considering their reliability or cross-modal differences. In contrast, LB-CIM first reduces unstable SAR responses, then exchanges information through a constrained cross-attention mechanism, and finally reorganizes the shared and complementary information after interaction. This sequence provides a more structured way of exploiting VIS–SAR complementarity. The qualitative comparisons support this interpretation: fewer missed detections and false positives are observed in bridge, harbor, dense-target, sparse-target, and mixed-scale scenes, although the magnitude of improvement varies with scene content and target distribution.

The complexity results also clarify the role of the low-bandwidth design. LB-CIM does not achieve its performance gain by substantially increasing model scale. Compared with computationally heavier multimodal fusion methods, the proposed model maintains a parameter count and computational cost close to the basic RGBT detector while obtaining higher detection accuracy. This behavior is consistent with the design of DLBCA, where channel projection and spatial reduction are applied to the cross-modal key and value representations rather than performing dense interaction over the complete feature space. Nevertheless, the current results only demonstrate the accuracy–complexity relationship at the model level; they do not by themselves establish that every individual reduction operation contributes equally to the final efficiency gain.

Several limitations remain. First, the experiments are conducted on two paired VIS–SAR datasets in which the two modalities are already spatially aligned. The robustness of LB-CIM to registration errors, large geometric misalignment, or temporally inconsistent observations has therefore not been fully evaluated. Second, the multi-scale discrepancy used in MCD serves as an indicator of local SAR instability rather than an explicit physical model of speckle noise, and its behavior may vary across sensors, resolutions, and imaging conditions. Third, although the cross-architecture experiments demonstrate consistent gains within the YOLO family, further evaluation on detectors with substantially different feature aggregation or query-based detection paradigms would provide a broader assessment of architectural generalization.

Future work will therefore focus on extending the proposed framework to multisensor VIS–SAR datasets with larger variations in spatial resolution, registration quality, and acquisition conditions. It would also be useful to incorporate uncertainty-aware or alignment-aware interaction mechanisms so that cross-modal fusion can adapt to imperfect correspondence between the two modalities. In addition, adaptive token reduction and content-dependent interaction bandwidth could further reduce redundant computation while preserving informative cross-modal responses. These directions may improve the robustness and general applicability of low-bandwidth VIS–SAR interaction without relying on a substantial increase in model complexity.

## 5. Conclusions

This paper presented LB-CIM, a low-bandwidth cross-modal interaction module for multimodal VIS–SAR object detection. The module follows a staged fusion strategy consisting of multi-scale consistency denoising (MCD), differential low-bandwidth cross-attention (DLBCA), and consensus-complementary enhancement (CCE). MCD reduces locally inconsistent SAR responses before multimodal interaction, DLBCA performs bidirectional information exchange using queries at the unreduced interaction-grid resolution and further spatially reduced key–value representations, and CCE reorganizes consensus and complementary cues after interaction. By integrating LB-CIM into multiple feature levels of a dual-branch detector, the proposed framework aims to exploit cross-modal complementarity without introducing dense interaction over the complete feature space.

Experiments on OGSOD-1.0 and SpaceNet6-OTD-Fog demonstrate the effectiveness of the proposed design. On OGSOD-1.0, LB-CIM improves the YOLO11-RGBT baseline by 4.8 percentage points in mAP_50_ and 4.7 percentage points in mAP_50:95_, reaching 95.1% and 56.8%, respectively. On SpaceNet6-OTD-Fog, the corresponding improvements are 2.7 and 2.0 percentage points, with mAP_50_ and mAP_50:95_ reaching 70.6% and 38.5%. The ablation experiments further show that MCD, DLBCA, and CCE provide complementary contributions, while evaluations on YOLOv5n-, YOLOv8n-, and YOLO11n-based RGBT detectors indicate that the effectiveness of LB-CIM is maintained across different YOLO architectures. The complete model contains 4.6 M parameters and requires 10.4 GFLOPs, providing a favorable balance between detection accuracy and computational complexity. Qualitative results also show fewer missed detections and false positives across different target categories and scene conditions.

Although the current experiments verify LB-CIM on two aligned VIS–SAR datasets, its behavior under larger registration errors, sensor differences, and more diverse acquisition conditions remains to be investigated. Future work will therefore consider alignment-aware cross-modal interaction, uncertainty-guided fusion, and adaptive token reduction to improve robustness under imperfect multimodal correspondence while further reducing redundant cross-modal computation. Overall, LB-CIM provides a compact multimodal sensing fusion strategy for heterogeneous VIS–SAR perception, with potential applicability to broader multi-sensor vision systems. 

## Figures and Tables

**Table 1:** Main architectural configuration of the YOLO11n-based LB-CIM detector for an input resolution of 640×640. The VIS and SAR backbone branches have identical topologies but independently learned parameters.

Level	Stride	VIS/SAR Feature	Input Ch.	Fusion	Output Feature
P3	8	128×80×80	128+128	LB-CIM	128×80×80
P4	16	128×40×40	128+128	LB-CIM	128×40×40
P5	32	256×20×20	256+256	LB-CIM	256×20×20

**Table 2:** Implementation configuration of DLBCA at the three pyramid levels. *C* denotes the channel dimension of the compact interaction features, $C_{a}$ is the attention channel dimension, *h* is the number of attention heads, *d* is the per-head dimension, and *r* denotes the spatial reduction factor of the key/value tokenizer.

Level	Interaction Grid	*C*	$C_{a}$	*h*	*d*	*r*	K/V Grid
P3	16×16	128	64	4	16	2	8×8
P4	16×16	128	64	4	16	2	8×8
P5	8×8	256	128	4	32	2	4×4

**Table 3:** Statistics of the VIS–SAR datasets used in the experiments.

Dataset	Training Pairs	Testing Pairs	Classes
OGSOD-1.0	14,664	3667	3
SpaceNet6-OTD-Fog	622	200	1

**Table 4:** Main training settings used in the experiments.

Setting	Configuration
Image size	640×640
Epochs	400
Batch size	64
Optimizer	SGD
Initial learning rate	0.01
Weight decay	$5\times 10^{-4}$
Learning-rate schedule	Cosine
Pretrained weights	No
Random seed	42
Final checkpoint	Last epoch (last.pt)

**Table 5:** Quantitative comparison of different methods on the OGSOD-1.0 dataset. The best and second-best detection results are highlighted in bold and underlined, respectively.

Modality	Method	Bridge ${\mathrm{AP}}_{50}$	Harbor ${\mathrm{AP}}_{50}$	Storage Tank ${\mathrm{AP}}_{50}$	P	R	${\mathrm{mAP}}_{50}$	${\mathrm{mAP}}_{50:95}$	Params (M)	GFLOPs
VIS	YOLOv5n	84.5	93.1	73.6	80.3	77.4	83.7	45.9	2.6	7.8
	YOLOv8n	84.9	94.4	75.2	83.5	80.1	84.8	46.3	3.2	8.9
	YOLO11n	86.3	95.6	76.8	85.8	82.3	86.2	48.2	2.6	6.5
	RT-DETR-L	89.8	94.3	80.4	86.5	85.0	88.2	49.7	32.0	103.4
SAR	YOLOv5n	81.4	92.2	71.8	79.5	75.7	81.8	45.2	2.6	7.8
	YOLOv8n	82.4	93.2	73.6	80.8	76.3	83.1	45.5	3.2	8.9
	YOLO11n	83.6	94.1	78.1	85.0	79.8	85.3	46.4	2.6	6.5
	RT-DETR-L	85.1	93.2	79.6	85.4	82.6	86.0	48.7	32.0	103.4
VIS–SAR	YOLO11-RGBT	90.8	97.4	82.8	88.9	85.0	90.3	52.1	3.7	9.3
	YOLO11-RGBT-Add	89.4	96.7	79.3	87.0	85.1	88.5	51.3	3.5	8.9
	SuperYOLO	93.8	96.9	90.4	86.2	86.8	93.7	53.8	4.8	56.2
	ICAFusion	94.7	97.5	88.0	88.4	86.5	93.4	54.1	20.2	29.8
	YOLO-CMFM	95.4	98.5	87.7	89.3	87.4	93.9	55.9	18.5	75.3
	**Ours**	**95.7**	**98.7**	**90.9**	**89.7**	**88.3**	**95.1**	**56.8**	4.6	10.4

**Table 6:** Quantitative comparison of different methods on the SpaceNet6-OTD-Fog dataset. The best and second-best detection results are highlighted in bold and underlined, respectively.

Modality	Method	P	R	${\mathrm{mAP}}_{50}$	${\mathrm{mAP}}_{50:95}$	Params (M)	GFLOPs
VIS	YOLO11n	77.7	55.1	62.8	34.6	2.6	6.5
SAR	YOLO11n	77.1	42.0	56.2	32.3	2.6	6.5
VIS–SAR	YOLO11-RGBT	81.4	58.2	67.9	36.5	3.7	9.3
	YOLO11-RGBT-Add	79.8	56.4	66.1	35.7	3.5	8.9
	SuperYOLO	84.8	62.7	69.2	36.9	4.8	56.2
	ICAFusion	83.2	61.3	68.8	37.2	20.2	29.8
	YOLO-CMFM	**85.6**	63.4	69.9	37.6	18.5	75.3
	**Ours**	85.3	**64.2**	**70.6**	**38.5**	4.6	10.4

**Table 7:** Ablation study of MCD, DLBCA, and CCE on the OGSOD-1.0 dataset. The best and second-best detection results are highlighted in bold and underlined, respectively.

MCD	DLBCA	CCE	P	R	${\mathrm{mAP}}_{50}$	${\mathrm{mAP}}_{50:95}$	Params (M)	GFLOPs
			88.9	85.0	90.3	52.1	3.7	9.3
✓			88.1	85.5	91.8	52.8	3.9	9.7
	✓		89.3	86.8	92.2	53.6	4.3	9.2
		✓	88.4	86.3	92.5	53.3	4.1	10.2
✓	✓		**90.0**	87.6	93.7	54.1	4.4	9.3
✓		✓	88.5	88.2	92.6	55.5	4.2	10.6
	✓	✓	88.7	87.9	94.7	56.1	4.6	9.9
✓	✓	✓	89.7	**88.3**	**95.1**	**56.8**	4.6	10.4

**Table 8:** Sensitivity analysis of pooling kernel configurations in MCD on OGSOD-1.0.

MCD Configuration	P (%)	R (%)	${\mathrm{mAP}}_{50}$ (%)	${\mathrm{mAP}}_{50:95}$ (%)
3×3	89.1	87.5	94.5	56.1
3×3+5×5 (Ours)	**89.7**	**88.3**	**95.1**	**56.8**
5×5+7×7	89.4	87.2	94.3	55.7

**Table 9:** Sensitivity analysis of spatial token reduction ratios in DLBCA on OGSOD-1.0.

Reduction Ratio	P (%)	R (%)	${\mathrm{mAP}}_{50}$ (%)	${\mathrm{mAP}}_{50:95}$ (%)	GFLOPs
1	**89.8**	**89.1**	**95.3**	**57.2**	12.9
2 (Ours)	89.7	88.3	95.1	56.8	10.4
4	89.2	87.4	94.3	55.7	**9.9**

**Table 10:** Comparison of different descriptor construction strategies in CCE on OGSOD-1.0.

Method	P (%)	R (%)	${\mathrm{mAP}}_{50}$ (%)	${\mathrm{mAP}}_{50:95}$ (%)	GFLOPs
Element-wise Product (Ours)	**89.7**	**88.3**	**95.1**	**56.8**	10.4
Cosine Similarity	89.3	87.6	94.5	56.1	10.4

**Table 11:** Mechanism-level ablation of DLBCA on the OGSOD-1.0 dataset. MCD, CCE, and the gate module are retained in all variants. “No” indicates that the gate output is bypassed, whereas “Yes” indicates that it is applied to the differential updates.

Variant	Value	Direction	Gate	${\mathrm{mAP}}_{50}$	${\mathrm{mAP}}_{50:95}$
A	Standard	Bidirectional	No	93.5	55.8
B	Differential	Bidirectional	No	94.0	56.1
C	Differential	SAR → VIS	Yes	94.4	56.4
D	Differential	VIS → SAR	Yes	93.9	56.2
E	Differential	Bidirectional	Yes	**95.1**	**56.8**

**Table 12:** Effect of MCD placement relative to cross-modal interaction on OGSOD-1.0.

Variant	Processing Order	${\mathrm{mAP}}_{50}$	${\mathrm{mAP}}_{50:95}$
A	DLBCA → CCE	94.7	56.1
B	DLBCA → MCD → CCE	94.5	55.8
C	MCD → DLBCA → CCE	**95.1**	**56.8**

**Table 13:** Effect of LB-CIM placement at different pyramid levels on OGSOD-1.0. A checkmark (✓) indicates that LB-CIM is applied at the corresponding pyramid level; an empty cell indicates that the baseline concatenation-based fusion is retained.

Variant	P3	P4	P5	${\mathrm{mAP}}_{50}$	${\mathrm{mAP}}_{50:95}$
A	✓			92.8	54.0
B		✓		92.1	53.5
C			✓	91.4	52.9
D	✓	✓		94.4	55.9
E		✓	✓	92.9	54.2
F	✓	✓	✓	**95.1**	**56.8**

**Table 14:** Generalization of LB-CIM across different YOLO architectures on OGSOD-1.0. Values in parentheses denote the performance gains over the corresponding RGBT baseline.

Architecture	Method	${\mathrm{mAP}}_{50}$	${\mathrm{mAP}}_{50:95}$	Params (M)	GFLOPs
YOLOv5n	RGBT	86.1	49.6	3.7	10.4
	RGBT + LB-CIM	**89.3 (+3.2)**	**52.4 (+2.8)**	4.7	11.4
YOLOv8n	RGBT	88.4	50.7	4.4	11.3
	RGBT + LB-CIM	**92.2 (+3.8)**	**53.9 (+3.2)**	5.2	12.2
YOLO11n	RGBT	90.3	52.1	3.7	9.3
	RGBT + LB-CIM	**95.1 (+4.8)**	**56.8 (+4.7)**	4.6	10.4

**Table 15:** Comparison between LB-CIM and a capacity-matched conventional baseline on OGSOD-1.0.

Method	Params (M)	GFLOPs	${\mathrm{mAP}}_{50}$ (%)	${\mathrm{mAP}}_{50:95}$ (%)
YOLO11-RGBT	3.70	9.30	90.3	52.1
YOLO11-RGBT-CM	4.63	10.91	92.8	54.0
LB-CIM (Ours)	4.60	10.40	**95.1**	**56.8**

**Table 16:** Measured inference efficiency of different VIS–SAR detection models on a single NVIDIA RTX 3090.

Method	Latency (ms)	Throughput (Images/s)	Peak GPU Memory (GiB)
YOLO11-RGBT	8.75	109.36	0.087
YOLO11-RGBT-Add	**8.70**	**115.04**	**0.080**
LB-CIM (Ours)	11.16	89.42	0.108

Note: All models are evaluated with a batch size of 1, a 640×640 four-channel input, and FP32 precision. Latency denotes the mean GPU forward-pass time measured using CUDA events. Throughput is measured over repeated forward passes and includes Python dispatch overhead. Data loading, preprocessing, and NMS are excluded. Peak GPU memory denotes the maximum memory allocated by PyTorch during the measured inference process.

**Table 17:** Quantitative detection error analysis on the OGSOD-1.0 test set. Changes are calculated as LB-CIM minus Baseline.

Method	TP	FP	FN	Precision (%)	Recall (%)	F1-Score (%)
Baseline	8251	2154	1363	79.30	85.82	82.43
LB-CIM	**8859**	**2070**	**755**	**81.06**	**92.15**	**86.25**
Change	**+608**	**−84**	**−608**	**+1.76**	**+6.33**	**+3.82**

**Table 18:** Detection error analysis under different scene-density groups on OGSOD-1.0. Groups are defined by the number of ground-truth objects per 640 × 640 image: sparse (1–2), medium-density (3–5), and dense (≥6).

Method	FP	FN	Precision (%)	Recall (%)	F1-Score (%)
*Sparse*					
Baseline	698	294	82.16	91.62	86.63
LB-CIM	656	143	83.69	95.92	89.39
Change	−42	−151	+1.53	+4.30	+2.76
*Medium-density*					
Baseline	552	448	82.13	84.99	83.54
LB-CIM	514	189	84.47	93.67	88.83
Change	−38	−259	+2.34	+8.68	+5.29
*Dense*					
Baseline	904	621	73.44	80.10	76.63
LB-CIM	900	423	74.99	86.45	80.31
Change	−4	−198	+1.55	+6.35	+3.68

## Data Availability

This study used the OGSOD-1.0 and SpaceNet6-OTD-Fog datasets. OGSOD-1.0 is publicly available at https://github.com/LICZ11/OGSOD-1.0-Dataset (accessed on 26 August 2026). SpaceNet6-OTD-Fog was derived from the publicly available SpaceNet6 multi-sensor dataset, which can be accessed at https://spacenet.ai/sn6-challenge/ (accessed on 26 August 2026). The original contributions presented in this study are included in the Supplementary Material.

## Supplementary Materials

The following supporting information can be downloaded at: https://www.mdpi.com/article/10.3390/s26217078/s1.
